# Safety Profile of a Cognitively Unimpaired Older Population with Elevated Cerebral Amyloid in a 4.5-Year Clinical Trial

**DOI:** 10.14283/jpad.2024.138

**Published:** 2024-07-24

**Authors:** Roy Yaari, K. C. Holdridge, M. Mancini, M. S. Rafii, M. Case, C. Battioui, J. R. Sims, P. S. Aisen, R. A. Sperling, A4 Study Team

**Affiliations:** 1 Eli Lilly and Company, Indianapolis, IN USA; 2 Alzheimer’s Therapeutic Research Institute, Keck School of Medicine, University of Southern California, San Diego, CA USA; 3 Center for Alzheimer Research and Treatment, Brigham and Women’s Hospital, Massachusetts General Hospital, Harvard Medical School, Boston, MA USA; 4 https://www.actcinfo.org/a4-study-team-lists/

**Keywords:** Amyloid-related imaging abnormalities, clinical trials, preclinical Alzheimer’s disease, safety monitoring

## Abstract

**Background:**

Preclinical Alzheimer’s disease is increasingly studied in clinical trials. Although safety signals are routinely monitored in clinical trial populations with Alzheimer’s disease, it can be challenging to identify new safety signals against background rates of age-related medical comorbidities.

**Objectives:**

To report detailed safety data from a cognitively unimpaired older population with evidence of elevated cerebral amyloid levels on amyloid positron emission tomography in the placebo arm of a Phase 3 clinical trial.

**Design:**

Phase 3, 4.5-year, multicenter, placebo-controlled trial.

**Setting:**

Placebo data from the Anti-Amyloid Treatment in Asymptomatic Alzheimer’s Disease (A4) study.

**Participants:**

Enrolled participants were aged 65–85 years with a global Clinical Dementia Rating score of 0, a Mini-Mental State Examination score of 2–-30, a Wechsler Memory Scale Logical Memory IIa (delayed recall) score of 6–18, and elevated brain amyloid levels on 18F-florbetapir positron emission tomography.

**Measurements:**

Study participants who received placebo were followed up with post-baseline safety measures. Assessments included review of concomitant medication and adverse events, the Columbia Suicide Severity Rating Scale, electrocardiograms, and neuroimaging (brain magnetic resonance imaging).

**Results:**

In total, 591 study participants (mean age [standard deviation] 71.9 [5.0] years) were assigned to and received placebo in the A4 study, and were followed up to 240 weeks. Participants were primarily White (93.9%) and from the United States (86.8%); 60.4% were women. The most common serious adverse events (incidence rate per 100 person-years) were pneumonia (incidence rate=0.4; 95% confidence interval=0.2–0.7) and atrial fibrillation (incidence rate=0.4; 95% confidence interval=0.2–0.7). The most common treatment-emergent adverse events were upper respiratory tract infection (incidence rate=10.9; 95% confidence interval=9.4–12.5), fall (incidence rate=7.7; 95% confidence interval=6.6–9.0), and nasopharyngitis (incidence rate=5.8; 95% confidence interval=4.8–6.9). The most common ischemia-related findings on magnetic resonance imaging were subcortical infarction (incidence rate=1.4; 95% confidence interval=1.0–2.0) and acute ischemia (incidence rate=0.6; 95% confidence interval=0.3–1.0). Emergent amyloid-related imaging abnormalities with hemosiderin deposition occurred in 32.8% of participants who received placebo; the primary factor associated with these events during the post-baseline period was the number of microhemorrhages at baseline (odds ratio=349.9; 95% confidence interval=247.6–494.4; adjusted p<0.001).

**Conclusion:**

Safety findings in the placebo-treated group from the A4 study provide a robust characterization of expected safety in a clinical trial population with preclinical Alzheimer’s disease. These results may provide context in planning future studies and safety evaluations during ongoing blinded studies in preclinical Alzheimer’s disease.

**Electronic Supplementary Material:**

Supplementary material is available in the online version of this article at 10.14283/jpad.2024.138.

## Introduction

Data suggest that evidence of elevated cerebral amyloid levels in cognitively unimpaired older persons represent a preclinical or asymptomatic stage of Alzheimer’s disease (AD) (1, 2), and these individuals may be at risk for cognitive decline (3, 4). A prospective cohort study of cognitively normal older individuals found a significant association between elevated brain amyloid levels at baseline and worse cognitive measures (1.59 points worse on the Preclinical Alzheimer Cognitive Composite and 0.56 points worse on the Mini-Mental State Examination) after 4 years (3). The Anti-Amyloid Treatment in Asymptomatic Alzheimer’s Disease (A4) study (NCT02008357) was a 4.5-year, Phase 3, double-blind, randomized, placebo-controlled trial demonstrating that solanezumab, an immunoglobulin G1 monoclonal antibody that binds to the mid-domain of the Aβ monomer, did not slow cognitive decline in individuals with preclinical AD compared with placebo (5, 6).

The population of individuals with preclinical AD is increasingly studied in clinical trials such as the AHEAD 3–45 and TRAILBLAZER-ALZ 3 studies (7, 8). In clinical trials of cognitively impaired AD populations, safety signals are routinely monitored through review of adverse events (AEs), laboratory tests, electrocardiograms, and brain imaging. However, it can be challenging to identify a new safety signal against background rates of age-related medical comorbidities (9). Reference data on background comorbidities in participants with preclinical AD can help speed identification of possible new safety signals in blinded clinical trials. This paper reports detailed safety data from a cognitively unimpaired older population with evidence of elevated cerebral amyloid levels on amyloid positron emission tomography (PET) from the placebo arm of the A4 study to aid safety monitoring and interpretation of ongoing and future preclinical AD studies.

## Methods

### Inclusion and exclusion criteria

The A4 study methods have been described previously (6). In brief, the A4 study enrolled participants at 67 sites in Australia, Canada, Japan, and the United States (6). Enrolled participants were aged 65–85 years with a global Clinical Dementia Rating score of 0 (ordinal scale, 0–3; 0 = no cognitive impairment and 3 = severe dementia), a Mini-Mental State Examination score of 25–30 (range, 0–30; lower scores indicate poorer cognitive performance), a Wechsler Memory Scale Logical Memory IIa (delayed recall) score of 6–18 (range, 0–25; lower scores indicate fewer details recalled), and elevated brain amyloid levels on 18F-florbetapir PET (6).

People with unstable medical conditions were excluded; participants with stable conditions such as hypertension, diabetes, hypercholesterolemia, and mild-to-moderate small-vessel ischemic disease were eligible (6). Additional safety exclusion criteria were history of human immunodeficiency virus infection, clinically significant multiple or severe drug allergies, post-treatment hypersensitivity reactions, schizophrenia, major depression or bipolar disorder in the past 2 years, and history within the past 5 years of a serious infectious disease affecting the brain, head trauma with protracted loss of consciousness, primary or recurrent malignant disease (with the exception of any in situ cancer that was appropriately treated), or chronic alcohol or drug abuse/dependence as defined by the most current version of the Diagnostic and Statistical Manual of Mental Disorders.

Participants were also excluded if they had a current or recent abnormal clinically significant laboratory result; electrocardiogram (ECG) revealed a clinically significant finding or Bazett’s corrected QT (QTcB) interval was >458 msec in males or >474 msec in females; or magnetic resonance imaging (MRI) revealed >4 hemosiderin deposits (definite microhemorrhages or areas of superficial siderosis) or any amyloid-related imaging abnormalities-edema/effusion (ARIA-E).

### Assessments

Participants in the placebo arm were followed up with post-baseline safety measures for up to 240 weeks in the double-blind placebo-controlled portion of the study. Concomitant medications and AEs were reviewed every 4 weeks. The Columbia Suicide Severity Rating Scale (C-SSRS) was administered approximately every 24 weeks. ECG was performed at weeks 12, 24, 48, 108, 168, and 240. Scheduled MRI was performed at weeks 12, 84, 168, and 240.

### Allowance of AD medications during the study

During initial screening, participants who used AD medications such as prescription acetylcholinesterase inhibitors and/or memantine were excluded from the study. Patients were allowed to start prescription acetylcholinesterase inhibitors and/or memantine during the course of the study with permission from the medical monitor.

### Statistical analysis

The population in this analysis included all participants randomized to the placebo arm who received at least one partial or full infusion. Only the placebo arm was used, as the treatment arm may include safety events related to the study drug.

Descriptive statistics were employed to summarize the safety data. To aid in the comparison of these safety data to findings from other clinical trials, rates are reported as incidence rates (IRs) per 100 person-years (PY). For MRI analysis, univariate logistic regression was performed with adjustment for false discovery rate to assess the association between baseline parameters and emergent amyloid-related imaging abnormalities-hemosiderin deposition (ARIA-H) among participants in the placebo group during the double-blind period of the study. Events occurring after the first administration of placebo were defined as treatment-emergent events. All statistical analyses were conducted using SAS version 9.4 except for baseline factors associated with ARIA-H, which used R software version 4.1.2.

### Coronavirus disease 2019 (COVID-19) pandemic disruption

The social distancing measures due to the COVID-19 pandemic necessitated a mid-trial hiatus in dose administration and testing. As a result, many visits were conducted later than the original target date. Therefore, some participants were observed beyond the scheduled 4.5-year period. Review of adverse events and concomitant medications continued every 4 weeks during the hiatus.

## Results

### Baseline characteristics

A total of 591 study participants (mean age [standard deviation] 71.9 [5.0] years) were assigned to and received placebo in the A4 study and were followed up with post-baseline safety measures for up to 240 weeks in the double-blind placebo period. Participants were primarily White (n=555; 93.9%) and from the United States (n=513; 86.8%), and 357 (60.4%) were women. Baseline characteristics of participants are summarized in Table 1.

**Table 1:** Baseline characteristics of participants in the placebo arm during the double-blind period of the A4 study (intent-to-treat population)

**Baseline characteristic**	Placebo (N=591)*
Female, n (%)	357 (60.4)
Age, years	
Mean (SD)	71.9 (5.0)
Median (range)	71.0 (65–86)
Age categories, n (%)	
65 to <75 years	439 (74.3)
75–85 years	152 (25.7)
Race, n (%)	
White	555 (93.9)
Black/African American	16 (2.7)
Asian	13 (2.2)
American Indian/Alaska Native	1 (0.2)
Multiple	3 (0.5)
Unknown	3 (0.5)
Ethnicity†, n (%)	
Hispanic or Latino	18 (3.0)
Not Hispanic or Latino	568 (96.1)
Not reported	5 (0.8)
Country/Region, n (%)	
United States	513 (86.8)
Australia	48 (8.1)
Canada	22 (3.7)
Japan	8 (1.4)
Mean BMI (SD), kg/m^2^	27.6 (5.2)*
APOE carriers, n (%)	347 (58.7)
Mean MMSE score (SD)	28.8 (1.2)
Mean CDR-SB (SD)	0.1 (0.2)
Mean CFI total score (SD)^§^	3.6 (3.3)^∥^
Mean ADCS-ADL-PQ (SD)	
Participant	43.1 (2.7)
Study Partner	43.5 (2.6)

ADCS-ADL-PQ = Alzheimer’s Disease Cooperative Study -Activity of Daily Living - Prevention Questionnaire; APOE = apolipoprotein E; BMI = Body Mass Index; CDR-SB = Clinical Dementia Rating-Sum of Boxes; CFI = Cognitive Function Index; MMSE = Mini-Mental State Examination; N = number of participants in the population; n = number of participants in the specified category; SD = Standard Deviation. * Participants with non-missing data; used as denominator unless otherwise specified. † Only includes responses from sites in the United States; n is the number of participants with a value of «Hispanic or Latino» or «Not Hispanic or Latino». ‡ N=590; § CFI total score includes both participant and partner score.

∥ N=589

At baseline, 585 participants (99.0%) had at least one preexisting condition. The most common preexisting conditions were hypertension (n=254; 43.0%), osteoarthritis (n=182; 30.8%), and gastroesophageal reflux disease (n=147; 24.9%). Preexisting conditions affecting ≥;10% of participants at baseline are listed in Supplementary Table 1. In all, 557 participants (94.2%) reported the concomitant use of at least one medication. The concomitant medications reported most at baseline were influenza vaccine (n=310; 52.5%), other viral vaccines (n=221; 37.4%), paracetamol (n=155; 26.2%), and ibuprofen (n=135; 22.8%). Concomitant medications received by ≥10% of participants at baseline are listed in Supplementary Table 1.

### Participant disposition and AEs

Of the 591 participants, 424 (71.7%; IR=17.4 [95% confidence interval {CI}: 15.7–19.1]) completed and 167 (28.3%; IR=6.8 [95% CI: 5.8–8.0]) discontinued the study. The primary or secondary reasons for study discontinuation were cited as “Withdrawal by participant” by 97 participants (16.4%; IR=4.0 [95% CI: 3.2–4.8]) and “Adverse event” by 46 (7.8%; IR=1.9 [95% CI: 1.4–2.5]). A full list of reasons for study discontinuation is included in Table 2. Among the participants who cited AEs as the primary reason for study discontinuation, atrial fibrillation was the only AE to affect more than one participant (n=2; 0.3%). The full list of AEs cited as the primary reason for study discontinuation is included in Table 2.

**Table 2:** Summary of participant disposition (intent-to-treat population)

**Parameter, n (%)**	**Placebo (N=591)**	**Incidence Rate per 100 PY (95% CI)**
Study Disposition		
Completed	424 (71.7)	17.4 (15.7, 19.1)
Discontinued	167 (28.3)	6.8 (5.8, 8.0)
Reasons for Study Discontinuation		
Withdrawal by participant	97 (16.4)	4.0 (3.2, 4.8)
AE	46 (7.8)	1.9 (1.4, 2.5)
Safety risk*	12 (2.0)	0.5 (0.3, 0.9)
Study partner unwilling or unable to participate	11 (1.9)	0.5 (0.2, 0.8)
Investigator recommendation	9 (1.5)	0.4 (0.2, 0.7)
COVID-19 Pandemic Disruption	8 (1.4)	0.3 (0.1, 0.6)
Lack of efficacy	7(1.2)	0.3 (0.1, 0.6)
Lost to follow-up	6(1.0)	0.2 (0.1, 0.5)
Death	5 (0.8)	0.2 (0.1, 0.5)
Non-compliance	2 (0.3)	0.1 (<0.1, 0.3)
Started prohibited medication	1 (0.2)	<0.1 (<0.1, 0.2)
Starting a new AD treatment	1 (0.2)	<0.1 (<0.1, 0.2)
Coordinating center request	1 (0.2)	<0.1 (<0.1, 0.2)
Other	32 (5.4)	1.3 (0.9, 1.9)
Other non-site clinician recommendation	3 (0.5)	0.1 (<0.1, 0.4)
AEs cited as primary reason for study discontinuation		
Atrial fibrillation	2 (0.3)	NA
Acetabulum fracture	1 (0.2)	NA
Acid-fast bacilli infection	1 (0.2)	NA
Alopecia	1 (0.2)	NA
ARIA-microhemorrhages and hemosiderin deposits	1 (0.2)	NA
Aortic dissection	1 (0.2)	NA
Atrioventricular block, second degree	1 (0.2)	NA
Cerebral artery embolism	1 (0.2)	NA
Cerebral hemorrhage	1 (0.2)	NA
Cerebrovascular accident	1 (0.2)	NA
Cholelithiasis	1 (0.2)	NA
Chronic lymphocytic leukemia	1 (0.2)	NA
Craniocerebral injury	1 (0.2)	NA
Diffuse large B-cell lymphoma	1 (0.2)	NA
Electrocardiogram PR prolongation	1 (0.2)	NA
Headache	1 (0.2)	NA
Hypertension	1 (0.2)	NA
Hypertensive urgency	1 (0.2)	NA
Influenza-like illness	1 (0.2)	NA
Infusion-related reaction	1 (0.2)	NA
Ischemic stroke	1 (0.2)	NA
Lymphoma	1 (0.2)	NA
Metastases to bone	1 (0.2)	NA
Non-small cell lung cancer	1 (0.2)	NA
Normal pressure hydrocephalus	1 (0.2)	NA
Pancreatic carcinoma	1 (0.2)	NA
Pancreatic carcinoma, metastatic	1 (0.2)	NA
Pancreatic carcinoma, stage III	1 (0.2)	NA
Papillary thyroid cancer	1 (0.2)	NA
Parkinsonism	1 (0.2)	NA
Psoriasis	1 (0.2)	NA
Rash	1 (0.2)	NA
Seizure	1 (0.2)	NA
Skin lesion	1 (0.2)	NA

Data are presented as n (%).; AD = Alzheimer’s disease; AE = adverse event; ARIA = amyloid-related imaging abnormality; CI = confidence interval; COVID-19 = coronavirus disease 2019; MRI = magnetic resonance imaging; N = number of participants in the population; n = number of participants in the specified category; NA = not applicable; PY = person-years. * Details of perceived safety risks for these 12 participants are as follows: Participant was advised by the Lilly Safety Team not to continue in the study if they would be unable to undergo MRI scans as safety assessments because of planned pacemaker placement (n=1); Participant had acute concern about the potential risk of microbleeds due to age and anticoagulant use and decided to withdraw from the study (n=1); Participant discontinued the study because of MRI safety risks (risk of cardiac event due to implanted pacemaker; n=1); Participant had a cranial infarct but was asymptomatic and was advised by their neurologist not to continue with the study (n=1); Participant discontinued per Lilly Safety Team decision (n=1); Participant discontinued due to AEs and additional safety risk with continuing infusions (n=1); Participant had been participating simultaneously in another clinical trial (n=1); Participant developed possible sensitivity to the drug, respiratory issues during the infusion (n=1); Participant started a new clinical trial medication, duvalumab, to treat worsening myelodysplastic syndrome (n=1); Participant discontinued due to multiple SAEs (n=1); Participant discontinued due to increased personal responsibilities, the time commitment required for study visits, and their perception of study drug safety risk (n=1); Participant discontinued due to safety reasons, although an MRI-compatible pacemaker was implanted (n=1).

Of the 591 participants, 158 (26.7%; IR=7.4 [95% CI: 6.3–8.7]) had serious AEs (SAEs) and 7 (1.2%) died (Table 3). The most common SAEs were pneumonia (n=9 [1.5%]; IR=0.4 [95% CI: 0.2–0.7]), atrial fibrillation (n=9 [1.5%]; IR = 0.4 [95% CI: 0.2–0.7]), myocardial infarction (n=7 [1.2%]; IR=0.3 [95% CI: 0.1–0.6]), osteoarthritis (n=7 [1.2%]; IR=0.3 [95% CI: 0.1–0.6]), and fall (n=6 [1.0%]; IR=0.2 [95% CI: 0.1–0.5]). A full list of observed SAEs is included in Table 3. Causes of death were pancreatic carcinoma (n=2), non-ST-elevation myocardial infarction (n=1), glioblastoma (n=1), car accident (n=1), malignant neoplasm of unknown origin (n=1), and myocardial infarction (n=1).

**Table 3:** AE overview and rates of SAEs among participants in the placebo arm during the double-blind period of the A4 study (safety population)

**Events**	Placebo (N=591)*	**Incidence Rate per 100 PY (95% CI)**
Discontinuations from the study primarily due to an AE	34 (5.8)	NA
Treatment-emergent AEs	577 (97.6)	NA
Deaths†	7 (1.2)	NA
Participants with ≥1 SAE	158 (26.7)	7.4 (6.3, 8.7)
Pneumonia	9 (1.5)	0.4 (0.2, 0.7)
Atrial fibrillation	9 (1.5)	0.4 (0.2, 0.7)
Myocardial infarction	7 (1.2)	0.3 (0.1, 0.6)
Osteoarthritis	7 (1.2)	0.3 (0.1, 0.6)
Fall	6 (1.0)	0.2 (0.1, 0.5)
Transient ischemic attack	4 (0.7)	0.2 (<0.1, 0.4)
Cellulitis	4 (0.7)	0.2 (<0.1, 0.4)
Sepsis	4 (0.7)	0.2 (<0.1, 0.4)
Acute myocardial infarction	4 (0.7)	0.2 (<0.1, 0.4)
Pelvic fracture	4 (0.7)	0.2 (<0.1, 0.4)
Arthritis	4 (0.7)	0.2 (<0.1, 0.4)
Pulmonary embolism	4 (0.7)	0.2 (<0.1, 0.4)
Syncope	3 (0.5)	0.1 (<0.1, 0.4)
Ischemic stroke	3 (0.5)	0.1 (<0.1, 0.4)
Subdural hematoma	3 (0.5)	0.1 (<0.1, 0.4)
Rib fracture	3 (0.5)	0.1 (<0.1, 0.4)
Humerus fracture	3 (0.5)	0.1 (<0.1, 0.4)
Rotator cuff syndrome	3 (0.5)	0.1 (<0.1, 0.4)
Deep vein thrombosis	3 (0.5)	0.1 (<0.1, 0.4)
Breast cancer	2 (0.3)	0.1 (<0.1, 0.3)
Pancreatic carcinoma	2 (0.3)	0.1 (<0.1, 0.3)
Cerebrovascular accident	2 (0.3)	0.1 (<0.1, 0.3)
Carotid artery stenosis	2 (0.3)	0.1 (<0.1, 0.3))
Urinary tract infection	2 (0.3)	0.1 (<0.1, 0.3)
Appendicitis	2 (0.3)	0.1 (<0.1, 0.3)
Diverticulitis	2 (0.3)	0.1 (<0.1, 0.3)
Diverticulitis intestinal perforated	2 (0.3)	0.1 (<0.1, 0.3)
Sinus node dysfunction	2 (0.3)	0.1 (<0.1, 0.3)
Hip fracture	2 (0.3)	0.1 (<0.1, 0.3)
Road traffic accident	2 (0.3)	0.1 (<0.1, 0.3)
Arthralgia	2 (0.3)	0.1 (<0.1, 0.3))
Intervertebral disc protrusion	2 (0.3)	0.1 (<0.1, 0.3)
Lumbar spinal stenosis	2 (0.3)	0.1 (<0.1, 0.3)
Aortic aneurism	2 (0.3)	0.1 (<0.1, 0.3)
Aortic dissection	2 (0.3)	0.1 (<0.1, 0.3)
Urinary retention	2 (0.3)	0.1 (<0.1, 0.3)
Vertigo	2 (0.3)	0.1 (<0.1, 0.3)
Chest pain	2 (0.3)	0.1 (<0.1, 0.3)
Prostate cancer‡	1 (0.4)	0.1 (<0.1, 0.6)
Invasive ductal breast carcinoma	1 (0.2)	<0.1 (<0.1, 0.2)
Bladder transitional cell carcinoma	1 (0.2)	<0.1 (<0.1, 0.2)
Diffuse large B-cell lymphoma	1 (0.2)	<0.1 (<0.1, 0.2)
Squamous cell carcinoma	1 (0.2)	<0.1 (<0.1, 0.2)
Angiomyolipoma	1 (0.2)	<0.1 (<0.1, 0.2)
Benign neoplasm of the bladder	1 (0.2)	<0.1 (<0.1, 0.2)
Chronic lymphocytic leukemia	1 (0.2)	<0.1 (<0.1, 0.2)
Glioblastoma	1 (0.2)	<0.1 (<0.1, 0.2)
Invasive breast carcinoma	1 (0.2)	<0.1 (<0.1, 0.2)
Lymphoma	1 (0.2)	<0.1 (<0.1, 0.2)
Malignant melanoma	1 (0.2)	<0.1 (<0.1, 0.2)
Metastases to bone	1 (0.2)	<0.1 (<0.1, 0.2)
Non-small cell lung cancer	1 (0.2)	<0.1 (<0.1, 0.2)
Ovarian cancer metastatic^6^	1 (0.3)	0.1 (<0.1, 0.4)
Pancreatic carcinoma metastatic	1 (0.2)	<0.1 (<0.1, 0.2)
Pancreatic carcinoma stage III	1 (0.2)	<0.1 (<0.1, 0.2)
Papillary thyroid cancer	1 (0.2)	<0.1 (<0.1, 0.2)
Parathyroid tumor benign	1 (0.2)	<0.1 (<0.1, 0.2)
Prostate cancer stage II‡	1 (0.4)	0.1 (<0.1, 0.6)
Renal cancer	1 (0.2)	<0.1 (<0.1, 0.2)
Transient global amnesia	1 (0.2)	<0.1 (<0.1, 0.2)
Ataxia	1 (0.2)	<0.1 (<0.1, 0.2)
Cerebral hemorrhage	1 (0.2)	<0.1 (<0.1, 0.2)
Lumbar radiculopathy	1 (0.2)	<0.1 (<0.1, 0.2)
Presyncope	1 (0.2)	<0.1 (<0.1, 0.2)
Sciatica	1 (0.2)	<0.1 (<0.1, 0.2)
Subarachnoid hemorrhage	1 (0.2)	<0.1 (<0.1, 0.2)
Cerebral artery embolism	1 (0.2)	<0.1 (<0.1, 0.2)
Cerebrospinal fluid leakage	1 (0.2)	<0.1 (<0.1, 0.2)
Embolic stroke	1 (0.2)	<0.1 (<0.1, 0.2)
Hemorrhage intracranial	1 (0.2)	<0.1 (<0.1, 0.2)
Headache	1 (0.2)	<0.1 (<0.1, 0.2)
Loss of consciousness	1 (0.2)	<0.1 (<0.1, 0.2)
Migraine with aura	1 (0.2)	<0.1 (<0.1, 0.2)
Myelopathy	1 (0.2)	<0.1 (<0.1, 0.2)
Parkinsonism	1 (0.2)	<0.1 (<0.1, 0.2)
Seizure	1 (0.2)	<0.1 (<0.1, 0.2)
Status epilepticus	1 (0.2)	<0.1 (<0.1, 0.2)
VIth nerve paralysis	1 (0.2)	<0.1 (<0.1, 0.2)
Escherichia bacteremia	1 (0.2)	<0.1 (<0.1, 0.2)
Escherichia urinary tract infection	1 (0.2)	<0.1 (<0.1, 0.2)
Urosepsis	1 (0.2)	<0.1 (<0.1, 0.2)
Campylobacter gastroenteritis	1 (0.2)	<0.1 (<0.1, 0.2)
Infection	1 (0.2)	<0.1 (<0.1, 0.2)
Influenza	1 (0.2)	<0.1 (<0.1, 0.2)
Labyrinthitis	1 (0.2)	<0.1 (<0.1, 0.2)
Localized infection	1 (0.2)	<0.1 (<0.1, 0.2)
Parainfluenza virus infection	1 (0.2)	<0.1 (<0.1, 0.2)
Peritonsillar abscess	1 (0.2)	<0.1 (<0.1, 0.2)
Rocky mountain spotted fever	1 (0.2)	<0.1 (<0.1, 0.2)
Streptococcal bacteremia	1 (0.2)	<0.1 (<0.1, 0.2)
Streptococcal sepsis	1 (0.2)	<0.1 (<0.1, 0.2)
Viral labyrinthitis	1 (0.2)	<0.1 (<0.1, 0.2)
Atrial flutter	1 (0.2)	<0.1 (<0.1, 0.2)
Cardiac failure congestive	1 (0.2)	<0.1 (<0.1, 0.2)
Coronary artery disease	1 (0.2)	<0.1 (<0.1, 0.2)
Atrioventricular block second degree	1 (0.2)	<0.1 (<0.1, 0.2)
Coronary artery stenosis	1 (0.2)	<0.1 (<0.1, 0.2)
Mitral valve prolapse	1 (0.2)	<0.1 (<0.1, 0.2)
Pericardial effusion	1 (0.2)	<0.1 (<0.1, 0.2)
Supraventricular tachycardia	1 (0.2)	<0.1 (<0.1, 0.2)
Small intestinal obstruction	1 (0.2)	<0.1 (<0.1, 0.2)
Gastrointestinal hemorrhage	1 (0.2)	<0.1 (<0.1, 0.2)
Hiatus hernia	1 (0.2)	<0.1 (<0.1, 0.2)
Large intestine polyp	1 (0.2)	<0.1 (<0.1, 0.2)
Diverticular perforation	1 (0.2)	<0.1 (<0.1, 0.2)
Duodenal ulcer	1 (0.2)	<0.1 (<0.1, 0.2)
Duodenal ulcer hemorrhage	1 (0.2)	<0.1 (<0.1, 0.2)
Gastric polyps	1 (0.2)	<0.1 (<0.1, 0.2)
Incarcerated inguinal hernia	1 (0.2)	<0.1 (<0.1, 0.2)
Inguinal hernia	1 (0.2)	<0.1 (<0.1, 0.2)
Inguinal hernia strangulated	1 (0.2)	<0.1 (<0.1, 0.2)
Intestinal ischemia	1 (0.2)	<0.1 (<0.1, 0.2)
Esophageal ulcer	1 (0.2)	<0.1 (<0.1, 0.2)
Esophagitis	1 (0.2)	<0.1 (<0.1, 0.2)
Upper gastrointestinal hemorrhage	1 (0.2)	<0.1 (<0.1, 0.2)
Craniocerebral injury	1 (0.2)	<0.1 (<0.1, 0.2)
Radius fracture	1 (0.2)	<0.1 (<0.1, 0.2)
Ulna fracture	1 (0.2)	<0.1 (<0.1, 0.2)
Wrist fracture	1 (0.2)	<0.1 (<0.1, 0.2)
Acetabulum fracture	1 (0.2)	<0.1 (<0.1, 0.2)
Clavicle fracture	1 (0.2)	<0.1 (<0.1, 0.2)
Contusion	1 (0.2)	<0.1 (<0.1, 0.2)
Femur fracture	1 (0.2)	<0.1 (<0.1, 0.2)
Foreign body in the gastrointestinal tract	1 (0.2)	<0.1 (<0.1, 0.2)
Head injury	1 (0.2)	<0.1 (<0.1, 0.2)
Lumbar vertebral fracture	1 (0.2)	<0.1 (<0.1, 0.2)
Patella fracture	1 (0.2)	<0.1 (<0.1, 0.2)
Procedural pain	1 (0.2)	<0.1 (<0.1, 0.2)
Spinal fracture	1 (0.2)	<0.1 (<0.1, 0.2)
Spinal stenosis	1 (0.2)	<0.1 (<0.1, 0.2)
Muscle spasms	1 (0.2)	<0.1 (<0.1, 0.2)
Cervical spinal stenosis	1 (0.2)	<0.1 (<0.1, 0.2)
Myalgia	1 (0.2)	<0.1 (<0.1, 0.2)
Spondylolisthesis	1 (0.2)	<0.1 (<0.1, 0.2)
Prostatitis*	1 (0.4)	0.1 (<0.1, 0.6)
Acute respiratory failure	1 (0.2)	<0.1 (<0.1, 0.2)
Dyspnea	1 (0.2)	<0.1 (<0.1, 0.2)
Hypoxia	1 (0.2)	<0.1 (<0.1, 0.2)
Pleural effusion	1 (0.2)	<0.1 (<0.1, 0.2)
Hypotension	1 (0.2)	<0.1 (<0.1, 0.2)
Hypertension	1 (0.2)	<0.1 (<0.1, 0.2)
Peripheral artery occlusion	1 (0.2)	<0.1 (<0.1, 0.2)
Cholelithiasis	1 (0.2)	<0.1 (<0.1, 0.2)
Cholecystitis	1 (0.2)	<0.1 (<0.1, 0.2)
Cholecystitis acute	1 (0.2)	<0.1 (<0.1, 0.2)
Hepatic cyst	1 (0.2)	<0.1 (<0.1, 0.2)
Hepatic cyst ruptured	1 (0.2)	<0.1 (<0.1, 0.2)
Hematuria	1 (0.2)	<0.1 (<0.1, 0.2)
Acute kidney injury	1 (0.2)	<0.1 (<0.1, 0.2)
Bladder prolapse	1 (0.2)	<0.1 (<0.1, 0.2)
Ureterolithiasis	1 (0.2)	<0.1 (<0.1, 0.2)
Urethral disorder	1 (0.2)	<0.1 (<0.1, 0.2)
Dehydration	1 (0.2)	<0.1 (<0.1, 0.2)
Hypokalemia	1 (0.2)	<0.1 (<0.1, 0.2)
Delirium	1 (0.2)	<0.1 (<0.1, 0.2)
Confusional state	1 (0.2)	<0.1 (<0.1, 0.2)
Alcoholism	1 (0.2)	<0.1 (<0.1, 0.2)
Chest discomfort	1 (0.2)	<0.1 (<0.1, 0.2)
Medical device site photosensitivity reaction	1 (0.2)	<0.1 (<0.1, 0.2)
Medical device site reaction	1 (0.2)	<0.1 (<0.1, 0.2)
Non-cardiac chest pain	1 (0.2)	<0.1 (<0.1, 0.2)
Closed fracture manipulation	1 (0.2)	<0.1 (<0.1, 0.2)
Knee arthroplasty	1 (0.2)	<0.1 (<0.1, 0.2)
Anemia	1 (0.2)	<0.1 (<0.1, 0.2)
Leukocytosis	1 (0.2)	<0.1 (<0.1, 0.2)
Cataract	1 (0.2)	<0.1 (<0.1, 0.2)

Percentages are based on the number of participants in the analysis population. Participants may be counted in more than one category. Note: Treatment-emergent adverse event is defined as an event that first occurred or worsened after first dose of study medication. Abbreviations: AE = adverse event; CI = confidence interval; N = number of participants in the analysis population; n = number of participants with events meeting specified criteria; NA = not applicable; PY = person-years; SAE = serious adverse event; TEAE = treatment-emergent adverse event. * Data are presented as n (%). † Deaths are also included as serious adverse events and discontinuations due to adverse events. ‡ Denominator adjusted because sex-specific event for males: N = 234. ^§^ Denominator adjusted because sex-specific event for females: N = 357.

In all, 577 participants (97.6%) had treatment-emergent AEs. The most common treatment-emergent AEs were upper respiratory tract infection (IR=10.9; 95% CI=9.4–12.5), fall (IR=7.7; 95% CI=6.6–9.0), nasopharyngitis (IR=5.8; 95% CI=4.8–6.9), headache (IR=5.6; 95% CI=4.6–6.7), and arthralgia (IR=5.5; 95% CI=4.6–6.6); all treatment-emergent AEs are listed in Supplementary Table 2.

### C-SSRS

Of the 583 participants with at least one post-baseline C-SSRS assessment, 44 (7.5%) experienced suicidal ideation (IR=1.9 [95% CI: 1.4–2.5]) and 6 (1.0%) experienced suicidal behavior (IR=0.2 [95% CI: 0.1–0.5]) during the study (Supplementary Table 3). The most common suicidal ideation events were wishing to be dead (n=28 [4.8%]; IR=1.2 [95% CI: 0.8–1.7]) and non-specific active suicidal thoughts (n=21 [3.6%]; IR=0.9 [95% CI: 0.5–1.3]). Suicidal behaviors included preparatory acts or behavior (n=5 [0.9%]; IR=0.2 [95% CI: 0.1–0.5]) and an aborted attempt (n=1 [0.2%]; IR=0.0 [95% CI: 0.0–0.2]).

### ECG

Treatment-emergent ECG abnormalities are summarized in Supplementary Table 4. The cardiac abnormalities that occurred most often during the doubleblind period of the study were low mean heart rate (n=71 [12.9%]; IR=3.2 [95% CI: 2.5–4.0]), high QT interval (n=64 [11.7%]; IR=2.8 [95% CI: 2.2–3.6]), high PR interval (n=34 [6.3%]; IR=1.4 [95% CI: 1.0–2.0]), and high QRS complex (n=18 [3.3%]; IR=0.7 [95% CI: 0.4–1.2]).

### Brain MRI

Of the 591 participants, 46 (7.8%; IR=2.0 [95% CI: 1.4–2.6]) had ischemia-related findings on MRI; the most common were subcortical infarction (n=34 [5.8%]; IR=1.4 [95% CI: 1.0–2.0]) and acute ischemia (n=14 [2.4%]; IR=0.6 [95% CI: 0.3–1.0]). ARIA-E occurred in 2 participants (0.3%; IR=0.1 [95% CI: 0.0–0.3]) and ARIA-H occurred in 194 participants (32.8%; IR=10.5 [95% CI: 9.1–12.1]); microhemorrhage occurred in 189 participants (32.0%; IR=10.2 [95% CI: 8.8–11.7]), and superficial siderosis occurred in 19 (3.2%; IR=0.8 [95% CI: 0.5–1.2]). With respect to apolipoprotein E (APOE) allele status, ARIA-H was observed in 57.4% of APOE ε4 homozygotes, 32.3% of APOE ε4 heterozygotes, and 28.7% of those with no APOE ε4 allele (Table 4). Treatment-emergent ischemia-related findings and incidence of ARIA-H by APOE allele status and genotype during the double-blind period of the study are summarized in Table 4.

**Table 4:** Treatment-emergent ischemia-related findings and incidence of ARIA-H among participants in the placebo arm during the double-blind period of the A4 study by safety brain MRI (safety population)

**Parameter**	**Placebo (N=591)**	**Incidence Rate per 100 PY (95% CI)**
Any ischemia-related findings, n (%)	46 (7.8)	2.0 (1.4, 2.6)
Large cortical infarction	1 (0.2)	<0.1 (<0.1, 0.2)
Small cortical infarction	9 (1.5)	0.4 (0.2, 0.7)
Subcortical infarction	34 (5.8)	1.4 (1.0, 2.0)
Acute ischemia	14 (2.4)	0.6 (0.3, 1.0)
ARIA-E, n (%)*	2 (0.3)	0.1 (<0.1, 0.3)
ARIA-H, n (%)^†^	194 (32.8)	10.5 (9.1, 12.1)
Microhemorrhage	189 (32.0)	10.2 (8.8, 11.7)
Superficial siderosis	19 (3.2)	0.8 (0.5, 1.2)
ARIA-H incidence by APOE allele status and genotype^‡^		
APOE ε4 carrier	124/347 (35.7)	NA
APOE ε4 heterozygote	97/300 (32.3)	NA
ε2/ε4	8/22 (36.4)	NA
ε3/ε4	89/278 (32.0)	NA
APOE ε4 homozygote	27/47 (57.4)	NA
APOE ε4 noncarrier	70/244 (28.7)	NA
ε2/ε2	0/0	NA
ε2/ε3	9/33 (27.3)	NA
ε3/ε3	61/211 (28.9)	NA

ARIA-H = amyloid-related imaging abnormalities–hemosiderin deposition; APOE = apolipoprotein E; CI = confidence interval; MRI = magnetic resonance imaging; N = number of participants in the safety population; n = number of participants within category; NA = not applicable; PY = person-years. * ARIA-E includes the following types of amyloid-related imaging abnormality: edema/effusion, brain edema. † ARIA-H includes the following types of amyloid-related imaging abnormality: microhemorrhages, brain stem microhemorrhage, cerebellar microhemorrhage, cerebral microhemorrhage, cerebral hemorrhage, and superficial siderosis of the central nervous system. ^‡^ For each category, the denominator is the number of participants with the respective genotype.

ARIA-H incidence by APOE allele status/genotype and use of concomitant antithrombotic medication is summarized in Table 5. ARIA-H were observed in approximately one-third (29.4%–35.3%) of participants irrespective of antithrombotic use. The percentage of participants with ARIA-H was numerically higher among those taking nonaspirin antiplatelet agents and thrombolytics, but the numbers in those groups were small (Table 5).

**Table 5:** Summary of ARIA-H incidence by use of concomitant antithrombotic medication among participants in the placebo arm during the double-blind period of the A4 study (safety population)

	**Concomitant Medication**						
	**No Antithrombotics (N=245)**	**Antithrombotic (N=346)**	**Type of Antithrombotic**				
			**Aspirin (N=326)**	**Nonaspirin Antiplatelet (N=33)**	**Anticoagulant (N=97)**	**Anticoagulant or Antiplatelet (N=346)**	**Thrombolytics (N=2)**
ARIA-H based on MRI, n (%)*	72 (29.4)	122 (35.3)	113 (34.7)	17 (51.5)	35 (36.1)	122 (35.3)	1 (50.0)
Microhemorrhage	72 (29.4)	117 (33.8)	108 (33.1)	16 (48.5)	35 (36.1)	117 (33.8)	1 (50.0)
Superficial siderosis	7 (2.9)	12 (3.5)	13 (4.0)	2 (6.1)	2 (2.1)	12 (3.5)	0
APOE ε4 carrier†	48/147 (32.7)	76/200 (38.0)	71/188 (37.8)	13/21 (61.9)	22/57 (38.6)	76/200 (38.0)	1/2 (50.0)
APOE ε4 heterozygote	38/128 (29.7)	59/172 (34.3)	55/162 (34.0)	8/16 (50.0)	18/48 (37.5)	59/172 (34.3)	1/2 (50.0)
ε2/ε4	2/9 (22.2)	6/13 (46.2)	6/11 (54.5)	1/1 (100)	1/4 (25.0)	6/13 (46.2)	0/0
ε3/ε4	36/119 (30.3)	53/159 (33.3)	49/151 (32.5)	7/15 (46.7)	17/44 (38.6)	53/159 (33.3)	1/2 (50.0)
APOE ε4 homozygote	10/19 (52.6)	17/28 (60.7)	16/26 (61.5)	5/5 (100)	4/9 (44.4)	17/28 (60.7)	0/0
APOE ε4 noncarrier*	24/98 (24.5)	46/146 (31.5)	42/138 (30.4)	4/12 (33.3)	13/40 (32.5)	46/146 (31.5)	0/0
ε2/ε2	0/0	0/0	0/0	0/0	0/0	0/0	0/0
ε2/v3	5/16 (31.3)	4/17 (23.5)	5/18 (27.8)	0/0	0/7 (0.0)	4/17 (23.5)	0/0
ε3/ε3	19/82 (23.2)	42/129 (32.6)	37/120 (30.8)	4/12 (33.3)	13/33 (39.4)	42/129 (32.6)	0/0

Abbreviations: ARIA-H = amyloid-related imaging abnormalities – hemorrhage; APOE = apolipoprotein E; MRI = magnetic resonance imaging; N = number of subjects in the safety population; n = number of subjects within category; TEAE = treatment-emergent adverse event. * Searches across ARIA-H adverse events did not find additional cases of ARIA-H. † Denominator is the number of subjects with respective genotype.

The most significant baseline factors associated with ARIA-H are detailed in Supplementary Table 5. The number of microhemorrhages at baseline emerged as the primary factor associated with ARIA-H events during the post-baseline period (odds ratio=349.9; 95% CI=247.6–494.4; adjusted p<0.001; Supplementary Table 5). A stark contrast was observed in the frequency of ARIA-H events between participants with microhemorrhages at baseline (98.4%) and those without (15.1%).

### Frequency of starting AD medication

Among the 591 participants, donepezil was started by 18 participants (3.0%), rivastigmine by 7 participants (1.2%), and memantine by 5 participants (0.8%) during the study.

## Discussion

Safety findings in the placebo-treated group from the A4 study provide a robust characterization of safety events in a clinical trial population with preclinical AD. Frequencies of treatment-emergent AEs were reported as events per 100 PY of observation to enable comparisons with studies of different durations.

Univariate analysis showed microhemorrhages at baseline to be the most significant baseline factor associated with emergent ARIA-H, far greater than other risk factors such as APOE4 status.

To provide context for the data collected from participants in the placebo arm of the A4 study, we compared the collected safety information to published safety information from symptomatic AD patients taking placebo in five 18-month AD dementia trials and from symptomatic AD patients in the Alzheimer’s Disease Neuroimaging Initiative study (referred to below as “reference populations” or “reference groups”) (9). The baseline characteristics of the A4 placebo population [mean age [SD] 71.9 [5.0] years; 60.4% women] were comparable to those of reference populations in Henley et al 2012 (9), in which mean ages ranged from 67.9 to 77.3 years and the percentage of women ranged from 47.9% to 61.0%.

At 28.3%, the frequency of overall discontinuations in the present placebo population was within the overall range of 8.2%–33.0% in the reference populations (9). Discontinuations due to AEs (5.8%; Table 3) were also comparable to those in the reference groups, which ranged from 2.7% to 11.6% in the included studies with this information (9). Among participants in the present placebo population who cited AEs as the primary reason for study discontinuation, atrial fibrillation was the only AE to affect more than one participant (n=2; 0.3%), and falls were not mentioned as an AE among participants who discontinued due to an AE. In comparison, falls were the most commonly reported AE among the AD dementia population reference groups, with rates ranging from 5.5% to 16.8% (9).

The observed frequency of deaths of 1.2% was also within the range reported by the reference groups (0%–2.4%) (9). However, the observed frequency of SAEs overall (26.7%) was higher than that reported by the reference groups (19.9%–21.2%) (9). Regarding specific SAEs, the most common SAEs observed in the A4 placebo group were pneumonia (1.5%), atrial fibrillation (1.5%), myocardial infarction (1.2%), osteoarthritis (1.2%), and fall (1.0%). In comparison, in reference groups reporting the frequency of individual SAEs, falls were the most common SAE reported by at least 2 patients (2.6%–4.0%), followed by chest pain (2.1%) and pneumonia (0.9%–2.1%) (9).

These analyses of the placebo group from the A4 trial had some limitations. These results will primarily be used in the comparison and contextualization of results from other clinical trials with similar study populations. They may not be generalizable to studies with different inclusion and exclusion criteria or geographic distribution or to the greater population outside the clinical trial setting. In addition, the A4 study participants were not representative of the clinical trial participants with preclinical AD in underrepresented minority groups. White matter hyperintensity was not quantified on brain MRI during the study and was not included in the analyses. Post-hoc quantification of white matter hyperintensity was conducted, and further analyses found an association with the number of incident microhemorrhages (10). Additionally, lockdowns due to the COVID-19 pandemic temporarily disrupted activities at the study sites in Australia, Canada, Japan, and the United States, although concomitant medications and AEs continued to be reviewed via telephone calls during the disruption. The prevalence of COVID-19 may have increased some safety observations such as upper respiratory adverse events, rendering some of this safety data less generalizable.

Although some evidence supports an increased risk of suicide attempts among older adults with a recent diagnosis of mild cognitive impairment or dementia (11), limited published research on suicidal ideation and behavior in preclinical AD populations prevents comparison with the frequency observed in the A4 study. Further analyses of A4 safety data will help characterize this risk in clinical trial populations with preclinical AD, including any potential effect on individuals’ behaviors after they learn of their elevated amyloid levels.

Overall, the findings appear to be consistent with expected background rates in this age group. These results may be used to provide context in planning future studies and for safety evaluations during ongoing blinded clinical trials in preclinical AD. However, this population may more closely match populations from future studies of preclinical AD and thus allow for more cogent comparisons.

### Electronic Supplementary Material

Supplementary material, approximately 142 KB.

## Data Availability

*Data availability:* Eli Lilly and Company provides access to all individual participant data collected during the trial, after anonymization, with the exception of pharmacokinetic or genetic data. Data are available to request 6 months after the indication studied has been approved in the US and EU and after primary publication acceptance, whichever is later. No expiration date of data requests is currently set once data are made available. Access is provided after a proposal has been approved by an independent review committee identified for this purpose and after receipt of a signed data sharing agreement. Data and documents, including the study protocol, statistical analysis plan, clinical study report, and blank or annotated case report forms, will be provided in a secure data sharing environment. For details on submitting a request, see the instructions provided at www.vivli.org.

## Notes

*A4 ClinicalTrials.gov number:* NCT02008357

## Notes

*Ethical standards:* This study was conducted in accordance with consensus ethics principles derived from international ethics guidelines, including the Declaration of Helsinki and Council for International Organizations of Medical Sciences (CIOMS) International Ethical Guidelines, as well as the ICH GCP Guideline [E6] and applicable laws and regulations. Written informed consent was obtained from all trial participants before any procedures were conducted.
