# Supplementary material for: Safety Profile of a Cognitively Unimpaired Older Population with Elevated Cerebral Amyloid in a 4.5-Year Clinical Trial
Source: J Prev Alzheimers Dis. 2024 Jul 24;11(4):857–68. doi: 10.14283/jpad.2024.138 (PMC11266256; doi:10.14283/jpad.2024.138)
Supplement: Supplementary file 1 — Supplementary material, approximately 142 KB. [file mmc1.docx]

**Supplementary Material**

**Supplementary Table 1. Baseline medical history and most commonly received concomitant medications among participants in the placebo arm during the double-blind period of the A4 study (intent-to-treat population)**

| **Preexisting Conditions Affecting ≥10% of Participants at Baseline, n (%)** | **Placebo (N=591)** |
| --- | --- |
| Participants with ≥1 preexisting condition | 585 (99.0) |
| Hypertension | 254 (43.0) |
| Osteoarthritis | 182 (30.8) |
| Gastroesophageal reflux disease | 147 (24.9) |
| Benign prostatic hyperplasia* | 56 (23.9) |
| Hyperlipidemia | 139 (23.5) |
| Seasonal allergy | 137 (23.2) |
| Hypercholesterolemia | 118 (20.0) |
| Drug hypersensitivity | 115 (19.5) |
| Insomnia | 107 (18.1) |
| Hypothyroidism | 95 (16.1) |
| Depression | 83 (14.0) |
| Erectile dysfunction* | 32 (13.7) |
| Postmenopause^†^ | 48 (13.4) |
| Osteopenia | 70 (11.8) |
| Arthritis | 67 (11.3) |
| Back pain | 60 (10.2) |
| Cataract | 59 (10.0) |
| **Concomitant Medications Received by ≥10% of Participants at Baseline, n (%)** |  |
| Participants with ≥1 medication | 557 (94.2) |
| Influenza vaccine | 310 (52.5) |
| Other viral vaccines | 221 (37.4) |
| Paracetamol | 155 (26.2) |
| Ibuprofen | 135 (22.8) |
| Amoxicillin | 114 (19.3) |
| Varicella zoster vaccine | 103 (17.4) |
| Azithromycin | 96 (16.2) |
| Prednisone | 96 (16.2) |
| Cephalexin | 95 (16.1) |
| Acetylsalicylic acid | 86 (14.6) |
| Hydrocortisone | 74 (12.5) |
| Guaifenesin | 71 (12.0) |
| Ciprofloxacin | 67 (11.3) |
| Doxycycline | 66 (11.2) |
| Lidocaine | 63 (10.7) |

Data are presented as n (%).

N = number of participants in the population; n = number of participants in the specified category.

* Denominator adjusted because sex-specific event for males: N = 234.

^†^ Denominator adjusted because sex-specific event for females: N = 357.

**Supplementary Table 2. Incidence rates of treatment-emergent AEs ≥1 per 100 PY among participants in the placebo arm during the double-blind period of the A4 study by Preferred Term (safety population)**

| **Treatment-emergent AEs, Incidence Rate per 100 PY (95% CI)** | **Placebo**  **(N=591)** |  |
| --- | --- | --- |
| Upper respiratory tract infection | 10.898 (9.432, 12.526) |  |
| Fall | 7.729 (6.578, 9.023) |  |
| Nasopharyngitis | 5.769 (4.787, 6.893) |  |
| Headache | 5.596 (4.632, 6.702) |  |
| Arthralgia | 5.545 (4.593, 6.635) |  |
| Urinary tract infection | 4.275 (3.455, 5.232) |  |
| Back pain | 3.460 (2.735, 4.319) |  |
| Diarrhea | 3.459 (2.734, 4.317) |  |
| Cataract | 3.337 (2.625, 4.183) |  |
| Sinusitis | 3.299 (2.591, 4.142) |  |
| Osteoarthritis | 2.782 (2.138, 3.560) |  |
| Bronchitis | 2.751 (2.114, 3.519) |  |
| Dizziness | 2.724 (2.088, 3.492) |  |
| Hypertension | 2.605 (1.988, 3.353) |  |
| Contusion | 2.443 (1.846, 3.173) |  |
| Pain in extremity | 2.308 (1.729, 3.019) |  |
| Basal cell carcinoma | 2.289 (1.715, 2.994) |  |
| Skin laceration | 2.286 (1.712, 2.990) |  |
| Cough | 2.158 (1.602, 2.846) |  |
| Fatigue | 1.972 (1.444, 2.631) |  |
| Influenza | 1.855 (1.342, 2.498) |  |
| Rash | 1.669 (1.186, 2.281) |  |
| Ligament sprain | 1.707 (1.220, 2.325) |  |
| Cerebral microhemorrhage | 1.686 (1.205, 2.296) |  |
| Viral upper respiratory tract infection | 1.675 (1.191, 2.290) |  |
| Muscle strain | 1.578 (1.111, 2.175) |  |
| Depression | 1.614 (1.142, 2.216) |  |
| Vertigo | 1.218 (0.816, 1.749) |  |
| Arthritis | 1.569 (1.105, 2.163) |  |
| Gastroesophageal reflux disease | 1.624 (1.149, 2.229) |  |
| Insomnia | 1.527 (1.070, 2.114) |  |
| Cognitive disorder | 1.437 (0.995, 2.009) |  |
| Muscle spasms | 1.401 (0.964, 1.967) |  |
| Anxiety | 1.442 (0.999, 2.015) |  |
| Pneumonia | 1.394 (0.959, 1.957) |  |
| Benign prostatic hyperplasia* | 1.381 (0.735, 2.361) |  |
| Nausea | 1.310 (0.890, 1.860) |  |
| Squamous cell carcinoma | 1.265 (0.854, 1.806) |  |
| Cellulitis | 1.267 (0.855, 1.809) |  |
| Atrial fibrillation | 1.300 (0.883, 1.845) |  |
| Arthropod bite | 1.269 (0.856, 1.812) |  |
| Skin abrasion | 1.177 (0.782, 1.701) |  |
| Actinic keratosis | 1.175 (0.780, 1.698) |  |
| Tooth fracture | 1.181 (0.785, 1.707) |  |
| Dermatitis contact | 1.099 (0.718, 1.610) |  |
| Tooth abscess | 1.096 (0.716, 1.606) |  |
| Paresthesia | 1.049 (0.679, 1.549) |  |
| Seasonal allergy | 1.004 (0.643, 1.494) |  |
| Bursitis | 1.012 (0.648, 1.505) |  |
| Herpes zoster | 1.010 (0.647, 1.503) |  |
| Pruritus | 1.010 (0.647, 1.503) |  |
| Myalgia | 0.965 (0.612, 1.449) |  |
| Dyspnea | 0.922 (0.578, 1.396) |  |
| Sciatica | 0.921 (0.577, 1.394) |  |
| COVID-19 | 0.905 (0.567, 1.370) |  |
| Gastroenteritis | 0.882 (0.546, 1.348) |  |
| Dental caries | 0.880 (0.545, 1.345) |  |
| Osteoporosis | 0.877 (0.543, 1.341) |  |
| Blood creatine phosphokinase increased | 0.842 (0.515, 1.301) |  |
| Rotator cuff syndrome | 0.838 (0.512, 1.294) |  |
| Neck pain | 0.837 (0.511, 1.293) |  |
| Tinnitus | 0.836 (0.511, 1.291) |  |
| Conjunctivitis | 0.836 (0.510, 1.291) |  |
| Syncope | 0.793 (0.477, 1.238) |  |
| Balance disorder | 0.793 (0.477, 1.238) |  |
| Oropharyngeal pain | 0.792 (0.477, 1.237) |  |
| Edema peripheral | 0.790 (0.476, 1.234) |  |
| Meniscus injury | 0.753 (0.446, 1.190) |  |
| Constipation | 0.751 (0.445, 1.186) |  |
| Hypercholesterolemia | 0.751 (0.445, 1.187) |  |
| Erectile dysfunction* | 0.734 (0.295, 1.513) |  |
| Nephrolithiasis | 0.710 (0.413, 1.136) |  |
| Chest pain | 0.707 (0.412, 1.133) |  |
| Neuropathy peripheral | 0.707 (0.412, 1.133) |  |
| Osteopenia | 0.707 (0.412, 1.131) |  |
| Anemia | 0.706 (0.411, 1.131) |  |
| Tooth infection | 0.749 (0.444, 1.183) |  |
| Diverticulitis | 0.667 (0.381, 1.083) |  |
| Gastroenteritis viral | 0.665 (0.380, 1.080) |  |
| Asthma | 0.665 (0.380, 1.080) |  |
| Palpitations | 0.664 (0.380, 1.079) |  |
| Memory impairment | 0.663 (0.379, 1.077) |  |
| Blood pressure increased | 0.663 (0.379, 1.077) |  |
| Large intestine polyp | 0.625 (0.350, 1.031) |  |
| Localized infection | 0.625 (0.350, 1.030) |  |
| Dermatitis | 0.624 (0.349, 1.030) |  |
| Weight decreased | 0.624 (0.349, 1.029) |  |
| Rhinitis allergic | 0.623 (0.349, 1.028) |  |
| Skin lesion | 0.621 (0.348, 1.024) |  |
| Vomiting | 0.584 (0.319, 0.980) |  |
| Inguinal hernia | 0.582 (0.318, 0.976) |  |
| Limb injury | 0.581 (0.318, 0.975) |  |
| Abdominal pain | 0.581 (0.317, 0.974) |  |
| Rib fracture | 0.579 (0.317, 0.972) |  |
| Vaccination complication | 0.577 (0.316, 0.968) |  |
| Foot fracture | 0.541 (0.288, 0.925) |  |
| Infusion site extravasation | 0.540 (0.288, 0.924) |  |
| Dry eye | 0.539 (0.287, 0.922) |  |
| Amnesia | 0.539 (0.287, 0.922) |  |
| Macular degeneration | 0.538 (0.286, 0.919) |  |
| Diplopia | 0.538 (0.286, 0.920) |  |
| Prostate cancer* | 0.517 (0.168, 1.205) |  |
| Hematuria | 0.499 (0.258, 0.872) |  |
| Abdominal discomfort | 0.499 (0.258, 0.871) |  |
| Synovial cyst | 0.498 (0.257, 0.870) |  |
| Hyperlipidemia | 0.498 (0.257, 0.869) |  |
| Chest discomfort | 0.498 (0.257, 0.870) |  |
| Vitamin D deficiency | 0.497 (0.257, 0.868) |  |
| Pyrexia | 0.496 (0.256, 0.866) |  |
| Plantar fasciitis | 0.458 (0.229, 0.819) |  |
| Carpal tunnel syndrome | 0.458 (0.228, 0.819) |  |
| Trigger finger | 0.456 (0.228, 0.816) |  |
| Rhinorrhea | 0.456 (0.227, 0.815) |  |
| Vertigo positional | 0.455 (0.227, 0.814) |  |
| Hiatus hernia | 0.454 (0.227, 0.813) |  |
| C-reactive protein increased | 0.416 (0.199, 0.765) |  |
| Glucose tolerance impaired | 0.416 (0.199, 0.765) |  |
| Ear infection | 0.414 (0.199, 0.761) |  |
| Hypersensitivity | | 0.414 (0.198, 0.761) |
| Joint injury | | 0.414 (0.198, 0.761) |
| Seborrheic keratosis | | 0.414 (0.199, 0.762) |
| Urinary incontinence | | 0.414 (0.198, 0.761) |
| Hematoma | 0.413 (0.198, 0.760) |  |
| Tendonitis | 0.413 (0.198, 0.760) |  |
| Drug hypersensitivity | 0.413 (0.198, 0.760) |  |
| Abdominal pain upper | 0.412 (0.198, 0.758) |  |
| Gastritis | 0.373 (0.170, 0.708) |  |
| Onychomycosis | 0.373 (0.170, 0.708) |  |
| Obstructive sleep apnea syndrome | 0.372 (0.170, 0.707) |  |
| Coronary artery disease | 0.372 (0.170, 0.707) |  |
| Dyspepsia | 0.372 (0.170, 0.706) |  |
| Alopecia | 0.372 (0.170, 0.706) |  |
| Nasal congestion | 0.372 (0.170, 0.705) |  |
| Viral infection | 0.372 (0.170, 0.707) |  |
| Blepharitis | 0.372 (0.170, 0.706) |  |
| Blood cholesterol increased | 0.372 (0.170, 0.707) |  |
| Gingivitis | 0.372 (0.170, 0.707) |  |
| Peripheral swelling | 0.371 (0.170, 0.704) |  |
| Toothache | 0.371 (0.170, 0.704) |  |
| Eczema | 0.371 (0.170, 0.704) |  |
| Animal bite | 0.371 (0.170, 0.705) |  |
| Presyncope | 0.371 (0.170, 0.705) |  |
| Thyroid mass | 0.371 (0.170, 0.704) |  |
| Gout | 0.371 (0.169, 0.704) |  |
| Lower respiratory tract infection | 0.331 (0.143, 0.652) |  |
| Sleep apnea syndrome | 0.331 (0.143, 0.652) |  |
| Foot deformity | 0.331 (0.143, 0.652) |  |
| Tendon rupture | 0.331 (0.143, 0.652) |  |
| Sinus congestion | 0.330 (0.143, 0.651) |  |
| Tremor | 0.330 (0.143, 0.651) |  |
| Ligament rupture | 0.330 (0.143, 0.651) |  |
| Hemorrhoids | 0.330 (0.142, 0.650) |  |
| Transient ischemic attack | 0.330 (0.142, 0.650) |  |
| Bradycardia | 0.330 (0.142, 0.650) |  |
| Glaucoma | 0.330 (0.142, 0.650) |  |
| Mitral valve incompetence | 0.330 (0.142, 0.650) |  |
| Head injury | 0.330 (0.142, 0.650) |  |
| Hypotension | 0.330 (0.142, 0.650) |  |
| Ecchymosis | 0.330 (0.142, 0.650) |  |
| Dehydration | 0.329 (0.142, 0.649) |  |
| Wrist fracture | 0.329 (0.142, 0.649) |  |
| Prostatitis* | 0.310 (0.064, 0.906) |  |
| Flushing | 0.290 (0.116, 0.597) |  |
| Hypothyroidism | 0.289 (0.116, 0.596) |  |
| Deep vein thrombosis | 0.289 (0.116, 0.595) |  |
| Musculoskeletal chest pain | 0.289 (0.116, 0.595) |  |
| Rosacea | 0.289 (0.116, 0.596) |  |
| Diverticulum | 0.289 (0.116, 0.595) |  |
| Muscular weakness | 0.289 (0.116, 0.596) |  |
| Posterior capsule opacification | 0.289 (0.116, 0.595) |  |
| Vitreous floaters | 0.288 (0.116, 0.594) |  |
| Spinal osteoarthritis | 0.288 (0.116, 0.594) |  |
| Atrial flutter | 0.288 (0.116, 0.594) |  |
| Fungal infection | 0.288 (0.116, 0.594) |  |
| Hypoesthesia | 0.288 (0.116, 0.594) |  |
| Hypertonic bladder | 0.288 (0.116, 0.594) |  |
| Chills | 0.288 (0.116, 0.593) |  |
| Cystitis | 0.288 (0.116, 0.593) |  |
| Malaise | 0.288 (0.116, 0.593) |  |
| Pulmonary mass | 0.288 (0.116, 0.594) |  |
| Vitamin B12 deficiency | 0.288 (0.116, 0.593) |  |
| Food poisoning | 0.288 (0.116, 0.594) |  |
| Humerus fracture | 0.288 (0.116, 0.594) |  |
| Deafness bilateral | 0.288 (0.116, 0.594) |  |
| Myocardial infarction | 0.288 (0.116, 0.594) |  |
| Vulvovaginal mycotic infection^†^ | 0.275 (0.075, 0.703) |  |
| Oral herpes | 0.248 (0.091, 0.539) |  |
| Joint dislocation | 0.248 (0.091, 0.539) |  |
| Nerve compression | 0.248 (0.091, 0.540) |  |
| Adverse drug reaction | 0.248 (0.091, 0.539) |  |
| Ophthalmic migraine | 0.248 (0.091, 0.539) |  |
| Dizziness postural | 0.248 (0.091, 0.539) |  |
| Pain | 0.248 (0.091, 0.539) |  |
| Vision blurred | 0.247 (0.091, 0.539) |  |
| Abnormal dreams | 0.247 (0.091, 0.539) |  |
| Influenza-like illness | 0.247 (0.091, 0.538) |  |
| Precancerous skin lesion | 0.247 (0.091, 0.538) |  |
| Urticaria | 0.247 (0.091, 0.538) |  |
| Road traffic accident | 0.247 (0.091, 0.537) |  |
| Lethargy | 0.247 (0.091, 0.539) |  |
| Aortic stenosis | 0.247 (0.090, 0.537) |  |
| Deafness | 0.247 (0.091, 0.537) |  |
| Ingrowing nail | 0.247 (0.091, 0.538) |  |
| Vitreous detachment | 0.247 (0.091, 0.538) |  |
| Hyperkeratosis | 0.247 (0.091, 0.538) |  |
| Hyponatremia | 0.247 (0.091, 0.538) |  |
| Pharyngitis | 0.247 (0.091, 0.539) |  |
| Breast cancer | 0.247 (0.091, 0.537) |  |
| Fungal foot infection | 0.247 (0.091, 0.538) |  |
| Melanocytic nevus | 0.247 (0.091, 0.539) |  |
| Carotid artery stenosis | 0.247 (0.091, 0.537) |  |
| Decreased appetite | 0.247 (0.091, 0.537) |  |
| Exostosis | 0.247 (0.091, 0.538) |  |
| Pain in jaw | 0.247 (0.091, 0.539) |  |
| Wound | 0.247 (0.091, 0.539) |  |
| Cholelithiasis | 0.246 (0.090, 0.536) |  |
| Radius fracture | 0.246 (0.090, 0.536) |  |
| Prostatic specific antigen increased* | 0.207 (0.025, 0.746) |  |
| Musculoskeletal stiffness | 0.206 (0.067, 0.482) |  |
| Cerebral infarction | 0.206 (0.067, 0.482) |  |
| Hordeolum | 0.206 (0.067, 0.481) |  |
| Epistaxis | 0.206 (0.067, 0.481) |  |
| Cyst | 0.206 (0.067, 0.481) |  |
| Irritability | 0.206 (0.067, 0.481) |  |
| Gait disturbance | 0.206 (0.067, 0.480) |  |
| Procedural pain | 0.206 (0.067, 0.480) |  |
| Spinal stenosis | 0.206 (0.067, 0.480) |  |
| Visual impairment | 0.206 (0.067, 0.480) |  |
| Lower respiratory tract congestion | 0.206 (0.067, 0.480) |  |
| Confusional state | 0.206 (0.067, 0.480) |  |
| Dermal cyst | 0.206 (0.067, 0.482) |  |
| Lumbar spinal stenosis | 0.206 (0.067, 0.480) |  |
| Dry skin | 0.206 (0.067, 0.480) |  |
| Joint swelling | 0.206 (0.067, 0.481) |  |
| Conjunctival hemorrhage | 0.206 (0.067, 0.481) |  |
| Dysgeusia | 0.206 (0.067, 0.481) |  |
| Electrocardiogram QT prolonged | 0.206 (0.067, 0.481) |  |
| Lyme disease | 0.206 (0.067, 0.481) |  |
| Atrophic vulvovaginitis^†^ | 0.206 (0.042, 0.601) |  |
| Asthenia | 0.206 (0.067, 0.480) |  |
| Chronic obstructive pulmonary disease | 0.206 (0.067, 0.480) |  |
| Depressed mood | 0.206 (0.067, 0.480) |  |
| Epiretinal membrane | 0.206 (0.067, 0.481) |  |
| Hyperhidrosis | 0.206 (0.067, 0.481) |  |
| Esophageal stenosis | 0.206 (0.067, 0.481) |  |
| Hypocalcemia | 0.206 (0.067, 0.481) |  |
| Urine analysis abnormal | 0.206 (0.067, 0.481) |  |
| Malignant melanoma | 0.206 (0.067, 0.480) |  |
| Periodontitis | 0.206 (0.067, 0.480) |  |
| Renal cyst | 0.206 (0.067, 0.480) |  |
| Varicose vein | 0.206 (0.067, 0.480) |  |
| Periarthritis | 0.206 (0.067, 0.480) |  |
| Balanoposthitis* | 0.206 (0.025, 0.744) |  |
| Biopsy prostate* | 0.206 (0.025, 0.746) |  |
| Dementia Alzheimer's type | 0.205 (0.067, 0.479) |  |
| Rhinitis | 0.205 (0.067, 0.479) |  |
| Tooth extraction | 0.205 (0.067, 0.479) |  |
| Amyloid related imaging abnormality-microhemorrhages and hemosiderin deposits | 0.205 (0.067, 0.478) |  |
| Urinary retention | 0.205 (0.067, 0.479) |  |
| Intervertebral disc degeneration | 0.165 (0.045, 0.422) |  |
| Angina pectoris | 0.165 (0.045, 0.421) |  |
| Arthropod sting | 0.165 (0.045, 0.422) |  |
| Intervertebral disc protrusion | 0.165 (0.045, 0.421) |  |
| Upper-airway cough syndrome | 0.165 (0.045, 0.422) |  |
| Cervical radiculopathy | 0.165 (0.045, 0.421) |  |
| Eyelid ptosis | 0.165 (0.045, 0.421) |  |
| Musculoskeletal pain | 0.165 (0.045, 0.422) |  |
| Somnolence | 0.165 (0.045, 0.422) |  |
| Aphasia | 0.165 (0.045, 0.421) |  |
| Rash papular | 0.165 (0.045, 0.421) |  |
| Dyspnea exertional | 0.165 (0.045, 0.421) |  |
| Ventricular extrasystoles | 0.165 (0.045, 0.422) |  |
| Photopsia | 0.165 (0.045, 0.422) |  |
| Initial insomnia | 0.165 (0.045, 0.422) |  |
| Retinal tear | 0.165 (0.045, 0.422) |  |
| Migraine | 0.164 (0.045, 0.421) |  |
| Subdural hematoma | 0.164 (0.045, 0.420) |  |
| Pollakiuria | 0.164 (0.045, 0.421) |  |
| Acute sinusitis | 0.164 (0.045, 0.421) |  |
| Greater trochanteric pain syndrome | 0.164 (0.045, 0.421) |  |
| Sinus bradycardia | 0.164 (0.045, 0.421) |  |
| Non-cardiac chest pain | 0.164 (0.045, 0.420) |  |
| Acute myocardial infarction | 0.164 (0.045, 0.420) |  |
| Benign neoplasm of skin | 0.164 (0.045, 0.421) |  |
| Cardiac murmur | 0.164 (0.045, 0.421) |  |
| Hip fracture | 0.164 (0.045, 0.421) |  |
| Laryngitis | 0.164 (0.045, 0.420) |  |
| Otitis externa | 0.164 (0.045, 0.421) |  |
| Aortic aneurysm | 0.164 (0.045, 0.420) |  |
| Colitis microscopic | 0.164 (0.045, 0.420) |  |
| Infusion site bruising | 0.164 (0.045, 0.421) |  |
| Nocturia | 0.164 (0.045, 0.420) |  |
| Concussion | 0.164 (0.045, 0.421) |  |
| Ear discomfort | 0.164 (0.045, 0.420) |  |
| Lymphadenopathy | 0.164 (0.045, 0.421) |  |
| Superficial siderosis of central nervous system | 0.164 (0.045, 0.420) |  |
| Supraventricular tachycardia | 0.164 (0.045, 0.421) |  |
| Type 2 diabetes mellitus | 0.164 (0.045, 0.420) |  |
| Anal incontinence | 0.164 (0.045, 0.421) |  |
| Blood glucose increased | 0.164 (0.045, 0.421) |  |
| Coccydynia | 0.164 (0.045, 0.421) |  |
| Ischemic stroke | 0.164 (0.045, 0.421) |  |
| Paronychia | 0.164 (0.045, 0.421) |  |
| Retinal detachment | 0.164 (0.045, 0.421) |  |
| Leukocytosis | 0.164 (0.045, 0.420) |  |
| Pelvic fracture | 0.164 (0.045, 0.420) |  |
| Sepsis | 0.164 (0.045, 0.420) |  |
| Cerumen impaction | 0.164 (0.045, 0.421) |  |
| Deafness unilateral | 0.164 (0.045, 0.421) |  |
| Gastric polyps | 0.164 (0.045, 0.421) |  |
| Heart rate increased | 0.164 (0.045, 0.421) |  |
| Heart rate irregular | 0.164 (0.045, 0.421) |  |
| Labyrinthitis | 0.164 (0.045, 0.420) |  |
| Pulmonary embolism | 0.164 (0.045, 0.421) |  |
| Stress fracture | 0.164 (0.045, 0.420) |  |
| Vaginal infection^†^ | 0.137 (0.017, 0.495) |  |
| Vulvovaginal candidiasis^†^ | 0.137 (0.017, 0.495) |  |
| Dry mouth | 0.123 (0.025, 0.360) |  |
| Dysphagia | 0.123 (0.025, 0.360) |  |
| Irritable bowel syndrome | 0.123 (0.025, 0.361) |  |
| Rash pruritic | 0.123 (0.025, 0.360) |  |
| Tenosynovitis stenosans | 0.123 (0.025, 0.360) |  |
| Erythema | 0.123 (0.025, 0.360) |  |
| Eye infection | 0.123 (0.025, 0.360) |  |
| Pharyngitis streptococcal | 0.123 (0.025, 0.360) |  |
| Abdominal pain lower | 0.123 (0.025, 0.360) |  |
| Skin infection | 0.123 (0.025, 0.360) |  |
| Wound infection | 0.123 (0.025, 0.360) |  |
| Cardiac failure congestive | 0.123 (0.025, 0.360) |  |
| Chronic kidney disease | 0.123 (0.025, 0.360) |  |
| Liver function test increased | 0.123 (0.025, 0.360) |  |
| Procedural headache | 0.123 (0.025, 0.360) |  |
| Rash macular | 0.123 (0.025, 0.361) |  |
| Suicidal ideation | 0.123 (0.025, 0.360) |  |
| Aortic arteriosclerosis | 0.123 (0.025, 0.360) |  |
| Arrhythmia | 0.123 (0.025, 0.360) |  |
| Facial bones fracture | 0.123 (0.025, 0.360) |  |
| Flank pain | 0.123 (0.025, 0.361) |  |
| Onychoclasis | 0.123 (0.025, 0.361) |  |
| Post procedural infection | 0.123 (0.025, 0.361) |  |
| Atrioventricular block second degree | 0.123 (0.025, 0.361) |  |
| Bone pain | 0.123 (0.025, 0.361) |  |
| Diabetes mellitus | 0.123 (0.025, 0.361) |  |
| Throat irritation | 0.123 (0.025, 0.361) |  |
| Coronary artery occlusion | 0.123 (0.025, 0.361) |  |
| Ear swelling | 0.123 (0.025, 0.360) |  |
| Fractured sacrum | 0.123 (0.025, 0.360) |  |
| Fungal skin infection | 0.123 (0.025, 0.361) |  |
| Gingival recession | 0.123 (0.025, 0.359) |  |
| Hemangioma | 0.123 (0.025, 0.361) |  |
| Helicobacter infection | 0.123 (0.025, 0.360) |  |
| Hepatic cyst | 0.123 (0.025, 0.361) |  |
| Hypobarism | 0.123 (0.025, 0.360) |  |
| Infected dermal cyst | 0.123 (0.025, 0.360) |  |
| Meibomian gland dysfunction | 0.123 (0.025, 0.360) |  |
| Ocular hypertension | 0.123 (0.025, 0.360) |  |
| Pneumonia bacterial | 0.123 (0.025, 0.360) |  |
| Polyarthritis | 0.123 (0.025, 0.361) |  |
| Rash maculo-papular | 0.123 (0.025, 0.360) |  |
| SARS-CoV-2 test positive | 0.123 (0.025, 0.359) |  |
| Skin papilloma | 0.123 (0.025, 0.360) |  |
| Spinal pain | 0.123 (0.025, 0.360) |  |
| Spondylolisthesis | 0.123 (0.025, 0.359) |  |
| Superficial vein thrombosis | 0.123 (0.025, 0.360) |  |
| Tooth disorder | 0.123 (0.025, 0.360) |  |
| Urge incontinence | 0.123 (0.025, 0.360) |  |
| Infusion site reaction | 0.123 (0.025, 0.360) |  |
| Lumbar radiculopathy | 0.123 (0.025, 0.360) |  |
| Soft tissue injury | 0.123 (0.025, 0.360) |  |
| Agitation | 0.123 (0.025, 0.360) |  |
| Bladder prolapse | 0.123 (0.025, 0.360) |  |
| Dementia | 0.123 (0.025, 0.359) |  |
| Eye irritation | 0.123 (0.025, 0.360) |  |
| Eye pain | 0.123 (0.025, 0.360) |  |
| Gastrointestinal hemorrhage | 0.123 (0.025, 0.360) |  |
| Hepatic enzyme increased | 0.123 (0.025, 0.360) |  |
| Hot flush | 0.123 (0.025, 0.360) |  |
| Infusion-related reaction | 0.123 (0.025, 0.360) |  |
| Intraocular pressure increased | 0.123 (0.025, 0.360) |  |
| Lip injury | 0.123 (0.025, 0.360) |  |
| Loss of consciousness | 0.123 (0.025, 0.360) |  |
| Night sweats | 0.123 (0.025, 0.360) |  |
| Respiratory tract infection viral | 0.123 (0.025, 0.360) |  |
| Upper limb fracture | 0.123 (0.025, 0.360) |  |
| Aortic valve incompetence | 0.123 (0.025, 0.360) |  |
| Barrett's esophagus | 0.123 (0.025, 0.360) |  |
| Breast mass | 0.123 (0.025, 0.360) |  |
| Cerebellar infarction | 0.123 (0.025, 0.360) |  |
| Costochondritis | 0.123 (0.025, 0.360) |  |
| Dyslipidemia | 0.123 (0.025, 0.360) |  |
| Dysphonia | 0.123 (0.025, 0.360) |  |
| Head discomfort | 0.123 (0.025, 0.360) |  |
| Lacrimation increased | 0.123 (0.025, 0.360) |  |
| Lipoma | 0.123 (0.025, 0.360) |  |
| Oral candidiasis | 0.123 (0.025, 0.360) |  |
| Tachycardia | 0.123 (0.025, 0.360) |  |
| Tension headache | 0.123 (0.025, 0.360) |  |
| Tinea pedis | 0.123 (0.025, 0.360) |  |
| Transient global amnesia | 0.123 (0.025, 0.360) |  |
| Tricuspid valve incompetence | 0.123 (0.025, 0.360) |  |
| Ankle fracture | 0.123 (0.025, 0.360) |  |
| Atypical pneumonia | 0.123 (0.025, 0.360) |  |
| Craniocerebral injury | 0.123 (0.025, 0.360) |  |
| Deafness neurosensory | 0.123 (0.025, 0.360) |  |
| Fibromyalgia | 0.123 (0.025, 0.360) |  |
| Groin pain | 0.123 (0.025, 0.360) |  |
| Intermittent claudication | 0.123 (0.025, 0.360) |  |
| Musculoskeletal discomfort | 0.123 (0.025, 0.360) |  |
| Patella fracture | 0.123 (0.025, 0.360) |  |
| Piriformis syndrome | 0.123 (0.025, 0.360) |  |
| Reflux laryngitis | 0.123 (0.025, 0.360) |  |
| Skin injury | 0.123 (0.025, 0.360) |  |
| Parkinson's disease | 0.123 (0.025, 0.359) |  |
| Platelet count increased | 0.123 (0.025, 0.359) |  |
| Bacterial prostatitis* | 0.103 (0.003, 0.574) |  |
| Gynecomastia* | 0.103 (0.003, 0.573) |  |
| Hematospermia* | 0.103 (0.003, 0.572) |  |
| Hydrocele* | 0.103 (0.003, 0.573) |  |
| Prostate cancer stage II* | 0.103 (0.003, 0.573) |  |
| Prostate infection* | 0.103 (0.003, 0.574) |  |
| Testicular pain* | 0.103 (0.003, 0.573) |  |
| Sleep disorder | 0.082 (0.010, 0.297) |  |
| Urine leukocyte esterase positive | 0.082 (0.010, 0.296) |  |
| Neuralgia | 0.082 (0.010, 0.297) |  |
| Acute kidney injury | 0.082 (0.010, 0.296) |  |
| Bundle branch block right | 0.082 (0.010, 0.296) |  |
| Otitis media | 0.082 (0.010, 0.296) |  |
| Bone contusion | 0.082 (0.010, 0.297) |  |
| Cerebrovascular accident | 0.082 (0.010, 0.296) |  |
| Gamma-glutamyltransferase increased | 0.082 (0.010, 0.296) |  |
| Hand fracture | 0.082 (0.010, 0.297) |  |
| Hypoacusis | 0.082 (0.010, 0.297) |  |
| Micturition urgency | 0.082 (0.010, 0.296) |  |
| Polymyalgia rheumatica | 0.082 (0.010, 0.296) |  |
| Psoriasis | 0.082 (0.010, 0.296) |  |
| Respiratory tract infection | 0.082 (0.010, 0.297) |  |
| Cerebral ischemia | 0.082 (0.010, 0.296) |  |
| Corneal abrasion | 0.082 (0.010, 0.297) |  |
| Furuncle | 0.082 (0.010, 0.297) |  |
| Gastrointestinal viral infection | 0.082 (0.010, 0.297) |  |
| Hematochezia | 0.082 (0.010, 0.296) |  |
| Muscle rupture | 0.082 (0.010, 0.297) |  |
| Edema | 0.082 (0.010, 0.296) |  |
| Restless legs syndrome | 0.082 (0.010, 0.297) |  |
| Sinus node dysfunction | 0.082 (0.010, 0.296) |  |
| Skin ulcer | 0.082 (0.010, 0.296) |  |
| Abnormal loss of weight | 0.082 (0.010, 0.296) |  |
| Conjunctivitis allergic | 0.082 (0.010, 0.296) |  |
| Dermatitis allergic | 0.082 (0.010, 0.296) |  |
| Dupuytren's contracture | 0.082 (0.010, 0.296) |  |
| Dysplastic nevus | 0.082 (0.010, 0.296) |  |
| Dysuria | 0.082 (0.010, 0.297) |  |
| Essential tremor | 0.082 (0.010, 0.296) |  |
| Flatulence | 0.082 (0.010, 0.297) |  |
| Hypokalemia | 0.082 (0.010, 0.296) |  |
| Infusion site swelling | 0.082 (0.010, 0.296) |  |
| Joint effusion | 0.082 (0.010, 0.296) |  |
| Paresthesia oral | 0.082 (0.010, 0.297) |  |
| Postoperative wound infection | 0.082 (0.010, 0.296) |  |
| Scoliosis | 0.082 (0.010, 0.296) |  |
| Sinus headache | 0.082 (0.010, 0.297) |  |
| Stress urinary incontinence | 0.082 (0.010, 0.297) |  |
| Vitamin D decreased | 0.082 (0.010, 0.297) |  |
| Acrochordon | 0.082 (0.010, 0.297) |  |
| Arteriosclerosis | 0.082 (0.010, 0.297) |  |
| Burns second degree | 0.082 (0.010, 0.297) |  |
| Face injury | 0.082 (0.010, 0.297) |  |
| Fibula fracture | 0.082 (0.010, 0.297) |  |
| Gastric ulcer | 0.082 (0.010, 0.297) |  |
| Hepatic steatosis | 0.082 (0.010, 0.297) |  |
| Periorbital hematoma | 0.082 (0.010, 0.297) |  |
| Productive cough | 0.082 (0.010, 0.297) |  |
| Raynaud's phenomenon | 0.082 (0.010, 0.297) |  |
| Seborrheic dermatitis | 0.082 (0.010, 0.297) |  |
| Sensory disturbance | 0.082 (0.010, 0.297) |  |
| Abdominal distension | 0.082 (0.010, 0.297) |  |
| Anxiety disorder | 0.082 (0.010, 0.297) |  |
| Dysesthesia | 0.082 (0.010, 0.297) |  |
| Eye hemorrhage | 0.082 (0.010, 0.297) |  |
| Goiter | 0.082 (0.010, 0.297) |  |
| Hematocrit decreased | 0.082 (0.010, 0.297) |  |
| Lacunar infarction | 0.082 (0.010, 0.297) |  |
| Morton's neuralgia | 0.082 (0.010, 0.297) |  |
| Myocardial ischemia | 0.082 (0.010, 0.297) |  |
| Pericardial effusion | 0.082 (0.010, 0.297) |  |
| QRS axis abnormal | 0.082 (0.010, 0.297) |  |
| Sacroiliitis | 0.082 (0.010, 0.297) |  |
| Skin disorder | 0.082 (0.010, 0.297) |  |
| Tendon disorder | 0.082 (0.010, 0.297) |  |
| Anosmia | 0.082 (0.010, 0.296) |  |
| Clavicle fracture | 0.082 (0.010, 0.296) |  |
| *Escherichia* urinary tract infection | 0.082 (0.010, 0.296) |  |
| Eye allergy | 0.082 (0.010, 0.296) |  |
| Frequent bowel movements | 0.082 (0.010, 0.296) |  |
| Joint instability | 0.082 (0.010, 0.296) |  |
| Laboratory test abnormal | 0.082 (0.010, 0.296) |  |
| Lichen sclerosus | 0.082 (0.010, 0.296) |  |
| Nightmare | 0.082 (0.010, 0.296) |  |
| Esophagitis | 0.082 (0.010, 0.296) |  |
| Vestibular neuronitis | 0.082 (0.010, 0.296) |  |
| Weight increased | 0.082 (0.010, 0.296) |  |
| Appendicitis | 0.082 (0.010, 0.296) |  |
| Arteriosclerosis coronary artery | 0.082 (0.010, 0.296) |  |
| Bowel movement irregularity | 0.082 (0.010, 0.296) |  |
| Calculus bladder | 0.082 (0.010, 0.296) |  |
| Cerebellar microhemorrhage | 0.082 (0.010, 0.296) |  |
| Cerebral hemorrhage | 0.082 (0.010, 0.296) |  |
| Cholecystitis | 0.082 (0.010, 0.296) |  |
| Delusion | 0.082 (0.010, 0.296) |  |
| Dyspareunia | 0.082 (0.010, 0.296) |  |
| Eustachian tube dysfunction | 0.082 (0.010, 0.296) |  |
| Hemoptysis | 0.082 (0.010, 0.296) |  |
| Hallucination, auditory | 0.082 (0.010, 0.296) |  |
| Hip arthroplasty | 0.082 (0.010, 0.296) |  |
| Hyperkalemia | 0.082 (0.010, 0.296) |  |
| Infusion site pain | 0.082 (0.010, 0.296) |  |
| Joint stiffness | 0.082 (0.010, 0.296) |  |
| Keratitis | 0.082 (0.010, 0.296) |  |
| Ocular rosacea | 0.082 (0.010, 0.296) |  |
| Periodontal disease | 0.082 (0.010, 0.296) |  |
| Perioral dermatitis | 0.082 (0.010, 0.296) |  |
| Polyp | 0.082 (0.010, 0.296) |  |
| Pyuria | 0.082 (0.010, 0.296) |  |
| Skin cancer | 0.082 (0.010, 0.296) |  |
| Skin exfoliation | 0.082 (0.010, 0.296) |  |
| Spondylitis | 0.082 (0.010, 0.296) |  |
| Urinary tract discomfort | 0.082 (0.010, 0.296) |  |
| Visual acuity reduced | 0.082 (0.010, 0.296) |  |
| Vitiligo | 0.082 (0.010, 0.296) |  |
| Adrenal mass | 0.082 (0.010, 0.296) |  |
| Affect lability | 0.082 (0.010, 0.296) |  |
| Age-related macular degeneration | 0.082 (0.010, 0.297) |  |
| Ageusia | 0.082 (0.010, 0.296) |  |
| Angioedema | 0.082 (0.010, 0.296) |  |
| Aortic dilatation | 0.082 (0.010, 0.296) |  |
| Aortic dissection | 0.082 (0.010, 0.296) |  |
| Bereavement | 0.082 (0.010, 0.296) |  |
| Biliary colic | 0.082 (0.010, 0.296) |  |
| Blood creatinine increased | 0.082 (0.010, 0.296) |  |
| Bronchiolitis | 0.082 (0.010, 0.297) |  |
| Carotid arteriosclerosis | 0.082 (0.010, 0.297) |  |
| Clinical dementia rating scale score abnormal | 0.082 (0.010, 0.297) |  |
| Conduction disorder | 0.082 (0.010, 0.297) |  |
| Conjunctivitis bacterial | 0.082 (0.010, 0.296) |  |
| Corneal dystrophy | 0.082 (0.010, 0.296) |  |
| Cystitis interstitial | 0.082 (0.010, 0.296) |  |
| Dental prosthesis placement | 0.082 (0.010, 0.296) |  |
| Diabetic retinopathy | 0.082 (0.010, 0.297) |  |
| Disturbance in attention | 0.082 (0.010, 0.296) |  |
| Diverticulitis intestinal perforated | 0.082 (0.010, 0.296) |  |
| Epicondylitis | 0.082 (0.010, 0.297) |  |
| Eye hematoma | 0.082 (0.010, 0.297) |  |
| Feeling cold | 0.082 (0.010, 0.297) |  |
| Generalized anxiety disorder | 0.082 (0.010, 0.297) |  |
| Genital herpes | 0.082 (0.010, 0.296) |  |
| Hair texture abnormal | 0.082 (0.010, 0.297) |  |
| Hypoproteinemia | 0.082 (0.010, 0.296) |  |
| Hypoxia | 0.082 (0.010, 0.297) |  |
| Iliotibial band syndrome | 0.082 (0.010, 0.296) |  |
| Infusion site pruritus | 0.082 (0.010, 0.297) |  |
| Liver function test abnormal | 0.082 (0.010, 0.296) |  |
| Lung adenocarcinoma | 0.082 (0.010, 0.296) |  |
| Mitral valve prolapse | 0.082 (0.010, 0.296) |  |
| Myxoid cyst | 0.082 (0.010, 0.296) |  |
| Nasal obstruction | 0.082 (0.010, 0.296) |  |
| Neovascular age-related macular degeneration | 0.082 (0.010, 0.296) |  |
| Esophageal polyp | 0.082 (0.010, 0.297) |  |
| Otitis media acute | 0.082 (0.010, 0.297) |  |
| Pancreatic carcinoma | 0.082 (0.010, 0.296) |  |
| Paralysis | 0.082 (0.010, 0.296) |  |
| Parkinsonism | 0.082 (0.010, 0.296) |  |
| Peripheral arterial occlusive disease | 0.082 (0.010, 0.297) |  |
| Peripheral venous disease | 0.082 (0.010, 0.296) |  |
| Renal cancer | 0.082 (0.010, 0.296) |  |
| Retinal vein occlusion | 0.082 (0.010, 0.297) |  |
| Rotator cuff repair | 0.082 (0.010, 0.296) |  |
| Scratch | 0.082 (0.010, 0.296) |  |
| Skin irritation | 0.082 (0.010, 0.296) |  |
| Solar lentigo | 0.082 (0.010, 0.297) |  |
| Stress | 0.082 (0.010, 0.296) |  |
| Tenosynovitis | 0.082 (0.010, 0.296) |  |
| Thalamic infarction | 0.082 (0.010, 0.296) |  |
| Throat tightness | 0.082 (0.010, 0.296) |  |
| Tooth injury | 0.082 (0.010, 0.297) |  |
| Tympanic membrane perforation | 0.082 (0.010, 0.296) |  |
| Ulcer | 0.082 (0.010, 0.297) |  |
| Vaccination site reaction | 0.082 (0.010, 0.296) |  |
| Viral rash | 0.082 (0.010, 0.296) |  |
| Vaginal hemorrhage^†^ | 0.068 (0.002, 0.380) |  |
| Vaginal prolapse^†^ | 0.068 (0.002, 0.380) |  |
| Uterine prolapse^†^ | 0.068 (0.002, 0.380) |  |
| Cystocele^†^ | 0.068 (0.002, 0.380) |  |
| Uterine leiomyoma^†^ | 0.068 (0.002, 0.380) |  |
| Endometrial cancer^†^ | 0.068 (0.002, 0.380) |  |
| Endometrial dysplasia^†^ | 0.068 (0.002, 0.380) |  |
| Ovarian cancer metastatic^†^ | 0.068 (0.002, 0.380) |  |
| Urogenital atrophy^†^ | 0.068 (0.002, 0.380) |  |
| Vulvovaginal discomfort^†^ | 0.068 (0.002, 0.380) |  |
| Vulvovaginal dryness^†^ | 0.068 (0.002, 0.380) |  |
| Aspartate aminotransferase increased | 0.041 (0.001, 0.229) |  |
| Knee arthroplasty | 0.041 (0.001, 0.228) |  |
| Staphylococcal infection | 0.041 (0.001, 0.228) |  |
| Rheumatoid arthritis | 0.041 (0.001, 0.228) |  |
| Small intestinal obstruction | 0.041 (0.001, 0.228) |  |
| Spinal compression fracture | 0.041 (0.001, 0.228) |  |
| Supraventricular extrasystoles | 0.041 (0.001, 0.228) |  |
| Thrombocytopenia | 0.041 (0.001, 0.229) |  |
| Alanine aminotransferase increased | 0.041 (0.001, 0.229) |  |
| Allergy to arthropod bite | 0.041 (0.001, 0.228) |  |
| Atrioventricular block first degree | 0.041 (0.001, 0.228) |  |
| Eye contusion | 0.041 (0.001, 0.229) |  |
| Hallucination | 0.041 (0.001, 0.228) |  |
| Iron deficiency | 0.041 (0.001, 0.228) |  |
| Limb mass | 0.041 (0.001, 0.228) |  |
| Orthostatic hypotension | 0.041 (0.001, 0.229) |  |
| Temporomandibular joint syndrome | 0.041 (0.001, 0.228) |  |
| Thermal burn | 0.041 (0.001, 0.229) |  |
| Upper respiratory tract congestion | 0.041 (0.001, 0.229) |  |
| Bladder cancer | 0.041 (0.001, 0.228) |  |
| Blister | 0.041 (0.001, 0.228) |  |
| Bronchitis viral | 0.041 (0.001, 0.228) |  |
| Bundle branch block left | 0.041 (0.001, 0.228) |  |
| Cervical spinal stenosis | 0.041 (0.001, 0.228) |  |
| Chronic sinusitis | 0.041 (0.001, 0.228) |  |
| *Clostridium difficile* infection | 0.041 (0.001, 0.228) |  |
| Delirium | 0.041 (0.001, 0.228) |  |
| Disorientation | 0.041 (0.001, 0.229) |  |
| Electrocardiogram abnormal | 0.041 (0.001, 0.229) |  |
| Coordination abnormal | 0.041 (0.001, 0.229) |  |
| Change of bowel habit | 0.041 (0.001, 0.229) |  |
| Diverticulum intestinal | 0.041 (0.001, 0.229) |  |
| Hemoglobin decreased | 0.041 (0.001, 0.229) |  |
| Herpes simplex | 0.041 (0.001, 0.229) |  |
| Increased tendency to bruise | 0.041 (0.001, 0.229) |  |
| Pityriasis rosea | 0.041 (0.001, 0.229) |  |
| Skin discoloration | 0.041 (0.001, 0.229) |  |
| Sunburn | 0.041 (0.001, 0.229) |  |
| Thrombosis | 0.041 (0.001, 0.229) |  |
| Bladder transitional cell carcinoma | 0.041 (0.001, 0.229) |  |
| Blood pressure systolic increased | 0.041 (0.001, 0.229) |  |
| Blood thyroid-stimulating hormone increased | 0.041 (0.001, 0.229) |  |
| Blood urine present | 0.041 (0.001, 0.229) |  |
| Breast hyperplasia | 0.041 (0.001, 0.229) |  |
| Cardiac failure | 0.041 (0.001, 0.229) |  |
| Colorectal adenoma | 0.041 (0.001, 0.229) |  |
| Cyst rupture | 0.041 (0.001, 0.229) |  |
| Dacryostenosis acquired | 0.041 (0.001, 0.229) |  |
| Drug eruption | 0.041 (0.001, 0.229) |  |
| Drug withdrawal syndrome | 0.041 (0.001, 0.229) |  |
| Ear pain | 0.041 (0.001, 0.229) |  |
| Excessive cerumen production | 0.041 (0.001, 0.229) |  |
| Hypergammaglobulinaemia benign monoclonal | 0.041 (0.001, 0.229) |  |
| Hyperuricemia | 0.041 (0.001, 0.229) |  |
| Hyposmia | 0.041 (0.001, 0.229) |  |
| Lower respiratory tract infection viral | 0.041 (0.001, 0.229) |  |
| Miliaria | 0.041 (0.001, 0.229) |  |
| Oral infection | 0.041 (0.001, 0.229) |  |
| Pain of skin | 0.041 (0.001, 0.229) |  |
| Platelet count decreased | 0.041 (0.001, 0.229) |  |
| Polyuria | 0.041 (0.001, 0.229) |  |
| Post herpetic neuralgia | 0.041 (0.001, 0.229) |  |
| Renal impairment | 0.041 (0.001, 0.229) |  |
| Retinitis | 0.041 (0.001, 0.229) |  |
| Shoulder arthroplasty | 0.041 (0.001, 0.229) |  |
| Sinonasal papilloma | 0.041 (0.001, 0.229) |  |
| Folliculitis | 0.041 (0.001, 0.228) |  |
| Gastrointestinal disorder | 0.041 (0.001, 0.228) |  |
| Granuloma | 0.041 (0.001, 0.228) |  |
| Intestinal obstruction | 0.041 (0.001, 0.228) |  |
| Invasive ductal breast carcinoma | 0.041 (0.001, 0.228) |  |
| Malignant melanoma in situ | 0.041 (0.001, 0.228) |  |
| Middle insomnia | 0.041 (0.001, 0.228) |  |
| Peroneal nerve palsy | 0.041 (0.001, 0.228) |  |
| Plasma cell myeloma | 0.041 (0.001, 0.228) |  |
| Post-traumatic neck syndrome | 0.041 (0.001, 0.228) |  |
| Radiculopathy | 0.041 (0.001, 0.228) |  |
| Rash erythematous | 0.041 (0.001, 0.228) |  |
| Respiratory disorder | 0.041 (0.001, 0.228) |  |
| Tendon injury | 0.041 (0.001, 0.228) |  |
| Abdominal hernia | 0.041 (0.001, 0.228) |  |
| Abscess limb | 0.041 (0.001, 0.228) |  |
| Arthropathy | 0.041 (0.001, 0.228) |  |
| Ataxia | 0.041 (0.001, 0.228) |  |
| Axillary pain | 0.041 (0.001, 0.228) |  |
| Burns first degree | 0.041 (0.001, 0.228) |  |
| Colitis | 0.041 (0.001, 0.228) |  |
| Dental implantation | 0.041 (0.001, 0.228) |  |
| Dental restoration failure | 0.041 (0.001, 0.228) |  |
| Feeling abnormal | 0.041 (0.001, 0.228) |  |
| Glycosylated hemoglobin increased | 0.041 (0.001, 0.228) |  |
| Hydronephrosis | 0.041 (0.001, 0.228) |  |
| Hypertensive urgency | 0.041 (0.001, 0.228) |  |
| Inflammation | 0.041 (0.001, 0.228) |  |
| Infusion site hematoma | 0.041 (0.001, 0.228) |  |
| Injection site extravasation | 0.041 (0.001, 0.228) |  |
| Keratoacanthoma | 0.041 (0.001, 0.228) |  |
| Lumbosacral radiculopathy | 0.041 (0.001, 0.228) |  |
| Meralgia paraesthetica | 0.041 (0.001, 0.228) |  |
| Middle ear effusion | 0.041 (0.001, 0.228) |  |
| Neuroma | 0.041 (0.001, 0.228) |  |
| Nodule | 0.041 (0.001, 0.228) |  |
| Osteonecrosis | 0.041 (0.001, 0.228) |  |
| Pancreatitis | 0.041 (0.001, 0.228) |  |
| Parosmia | 0.041 (0.001, 0.228) |  |
| Pleural effusion | 0.041 (0.001, 0.228) |  |
| Post procedural complication | 0.041 (0.001, 0.228) |  |
| Procedural anxiety | 0.041 (0.001, 0.228) |  |
| Pterygium | 0.041 (0.001, 0.228) |  |
| Rectal hemorrhage | 0.041 (0.001, 0.228) |  |
| Skin fissures | 0.041 (0.001, 0.228) |  |
| Stomatitis | 0.041 (0.001, 0.228) |  |
| Subarachnoid hemorrhage | 0.041 (0.001, 0.228) |  |
| Thyroid cyst | 0.041 (0.001, 0.228) |  |
| Tinea infection | 0.041 (0.001, 0.228) |  |
| Trigeminal neuralgia | 0.041 (0.001, 0.228) |  |
| Vaccination site pain | 0.041 (0.001, 0.228) |  |
| Ventricular tachycardia | 0.041 (0.001, 0.228) |  |
| Abdominal tenderness | 0.041 (0.001, 0.228) |  |
| Abscess oral | 0.041 (0.001, 0.228) |  |
| Acute respiratory failure | 0.041 (0.001, 0.228) |  |
| Allergy to animal | 0.041 (0.001, 0.228) |  |
| Allergy to arthropod sting | 0.041 (0.001, 0.228) |  |
| Animal scratch | 0.041 (0.001, 0.228) |  |
| Atelectasis | 0.041 (0.001, 0.228) |  |
| Aura | 0.041 (0.001, 0.228) |  |
| Benign neoplasm of bladder | 0.041 (0.001, 0.228) |  |
| Bile duct stone | 0.041 (0.001, 0.228) |  |
| Biliary dilatation | 0.041 (0.001, 0.228) |  |
| Blepharoplasty | 0.041 (0.001, 0.228) |  |
| Blepharospasm | 0.041 (0.001, 0.228) |  |
| Blood alkaline phosphatase increased | 0.041 (0.001, 0.228) |  |
| Blood testosterone decreased | 0.041 (0.001, 0.228) |  |
| Blood urea increased | 0.041 (0.001, 0.228) |  |
| Bone graft | 0.041 (0.001, 0.228) |  |
| Candida infection | 0.041 (0.001, 0.228) |  |
| Cartilage injury | 0.041 (0.001, 0.228) |  |
| Cataract nuclear | 0.041 (0.001, 0.228) |  |
| Cerebral small vessel ischemic disease | 0.041 (0.001, 0.228) |  |
| Cholecystitis acute | 0.041 (0.001, 0.228) |  |
| Chronic lymphocytic leukemia | 0.041 (0.001, 0.228) |  |
| Coronary artery stenosis | 0.041 (0.001, 0.228) |  |
| Diarrhea infectious | 0.041 (0.001, 0.228) |  |
| Diffuse large B-cell lymphoma | 0.041 (0.001, 0.228) |  |
| Duodenal ulcer | 0.041 (0.001, 0.228) |  |
| Electrolyte imbalance | 0.041 (0.001, 0.228) |  |
| Emphysema | 0.041 (0.001, 0.228) |  |
| Endodontic procedure | 0.041 (0.001, 0.228) |  |
| Escherichia bacteremia | 0.041 (0.001, 0.228) |  |
| Eustachian tube obstruction | 0.041 (0.001, 0.228) |  |
| Eye injury | 0.041 (0.001, 0.228) |  |
| Facial paralysis | 0.041 (0.001, 0.228) |  |
| Fecaloma | 0.041 (0.001, 0.228) |  |
| Femur fracture | 0.041 (0.001, 0.228) |  |
| Fracture displacement | 0.041 (0.001, 0.228) |  |
| Gastroenteritis norovirus | 0.041 (0.001, 0.228) |  |
| Granuloma annulare | 0.041 (0.001, 0.228) |  |
| Hemorrhage | 0.041 (0.001, 0.228) |  |
| Hemorrhoidal hemorrhage | 0.041 (0.001, 0.228) |  |
| Heat exhaustion | 0.041 (0.001, 0.228) |  |
| Hypercalcemia | 0.041 (0.001, 0.228) |  |
| Hyperglycemia | 0.041 (0.001, 0.228) |  |
| Ill-defined disorder | 0.041 (0.001, 0.228) |  |
| Impaired fasting glucose | 0.041 (0.001, 0.228) |  |
| Infection | 0.041 (0.001, 0.228) |  |
| Infusion site erythema | 0.041 (0.001, 0.228) |  |
| Infusion site hemorrhage | 0.041 (0.001, 0.228) |  |
| International normalized ratio increased | 0.041 (0.001, 0.228) |  |
| Intervertebral disc displacement | 0.041 (0.001, 0.228) |  |
| Iritis | 0.041 (0.001, 0.228) |  |
| Leukopenia | 0.041 (0.001, 0.228) |  |
| Ligament injury | 0.041 (0.001, 0.228) |  |
| Limb crushing injury | 0.041 (0.001, 0.228) |  |
| Lower gastrointestinal hemorrhage | 0.041 (0.001, 0.228) |  |
| Lower urinary tract symptoms | 0.041 (0.001, 0.228) |  |
| Major depression | 0.041 (0.001, 0.228) |  |
| Mass | 0.041 (0.001, 0.228) |  |
| Medical device removal | 0.041 (0.001, 0.228) |  |
| Meningioma | 0.041 (0.001, 0.228) |  |
| Mental state changes | 0.041 (0.001, 0.228) |  |
| Mole excision | 0.041 (0.001, 0.228) |  |
| Multiple allergies | 0.041 (0.001, 0.228) |  |
| Myelodysplastic syndrome | 0.041 (0.001, 0.228) |  |
| Nasal injury | 0.041 (0.001, 0.228) |  |
| Neoplasm | 0.041 (0.001, 0.228) |  |
| Notalgia paraesthetica | 0.041 (0.001, 0.228) |  |
| Occult blood positive | 0.041 (0.001, 0.228) |  |
| Ocular discomfort | 0.041 (0.001, 0.228) |  |
| Ocular hyperemia | 0.041 (0.001, 0.228) |  |
| Open angle glaucoma | 0.041 (0.001, 0.228) |  |
| Oral fungal infection | 0.041 (0.001, 0.228) |  |
| Oral pain | 0.041 (0.001, 0.228) |  |
| Parathyroid tumor benign | 0.041 (0.001, 0.228) |  |
| Partial seizures | 0.041 (0.001, 0.228) |  |
| Peptic ulcer | 0.041 (0.001, 0.228) |  |
| Periodic limb movement disorder | 0.041 (0.001, 0.228) |  |
| Peripheral artery occlusion | 0.041 (0.001, 0.228) |  |
| Peripheral artery stenosis | 0.041 (0.001, 0.228) |  |
| Pharyngeal mass | 0.041 (0.001, 0.228) |  |
| Phlebitis | 0.041 (0.001, 0.228) |  |
| Post lumbar puncture syndrome | 0.041 (0.001, 0.228) |  |
| Pulmonary hypertension | 0.041 (0.001, 0.228) |  |
| Rectal polyp | 0.041 (0.001, 0.228) |  |
| Renal function test abnormal | 0.041 (0.001, 0.228) |  |
| Retinal degeneration | 0.041 (0.001, 0.228) |  |
| Retinal hemorrhage | 0.041 (0.001, 0.228) |  |
| Seizure | 0.041 (0.001, 0.228) |  |
| Sensory loss | 0.041 (0.001, 0.228) |  |
| Soft tissue mass | 0.041 (0.001, 0.228) |  |
| Spinal claudication | 0.041 (0.001, 0.228) |  |
| Spinal fracture | 0.041 (0.001, 0.228) |  |
| Streptococcal bacteremia | 0.041 (0.001, 0.228) |  |
| Tooth impacted | 0.041 (0.001, 0.228) |  |
| Traumatic hematoma | 0.041 (0.001, 0.228) |  |
| Ulna fracture | 0.041 (0.001, 0.228) |  |
| Umbilical hernia | 0.041 (0.001, 0.228) |  |
| Upper gastrointestinal hemorrhage | 0.041 (0.001, 0.228) |  |
| Urinary tract obstruction | 0.041 (0.001, 0.228) |  |
| Urine odor abnormal | 0.041 (0.001, 0.228) |  |
| Urosepsis | 0.041 (0.001, 0.228) |  |
| Vasculitis | 0.041 (0.001, 0.228) |  |
| Venous angioma of brain | 0.041 (0.001, 0.228) |  |
| Vertebral osteophyte | 0.041 (0.001, 0.229) |  |
| Visual field defect | 0.041 (0.001, 0.229) |  |
| White blood cell count decreased | 0.041 (0.001, 0.229) |  |
| White blood cell count increased | 0.041 (0.001, 0.228) |  |
| White matter lesion | 0.041 (0.001, 0.228) |  |
| Abnormal feces | 0.041 (0.001, 0.228) |  |
| Acetabulum fracture | 0.041 (0.001, 0.228) |  |
| Acid-fast bacilli infection | 0.041 (0.001, 0.228) |  |
| Alcohol intolerance | 0.041 (0.001, 0.228) |  |
| Alcoholism | 0.041 (0.001, 0.228) |  |
| Allergy to metals | 0.041 (0.001, 0.228) |  |
| Amaurosis fugax | 0.041 (0.001, 0.228) |  |
| Anal fungal infection | 0.041 (0.001, 0.228) |  |
| Anal skin tags | 0.041 (0.001, 0.229) |  |
| Androgenetic alopecia | 0.041 (0.001, 0.228) |  |
| Angiomyolipoma | 0.041 (0.001, 0.229) |  |
| Anorectal discomfort | 0.041 (0.001, 0.228) |  |
| Antinuclear antibody increased | 0.041 (0.001, 0.228) |  |
| Aortic valve disease | 0.041 (0.001, 0.228) |  |
| Arterial bruit | 0.041 (0.001, 0.229) |  |
| Artificial crown procedure | 0.041 (0.001, 0.228) |  |
| Asthenopia | 0.041 (0.001, 0.229) |  |
| Atheroembolism | 0.041 (0.001, 0.228) |  |
| Atrial septal defect | 0.041 (0.001, 0.228) |  |
| Atrial tachycardia | 0.041 (0.001, 0.228) |  |
| Attention deficit hyperactivity disorder | 0.041 (0.001, 0.228) |  |
| Babesiosis | 0.041 (0.001, 0.228) |  |
| Bacterial food poisoning | 0.041 (0.001, 0.228) |  |
| Bacterial rhinitis | 0.041 (0.001, 0.228) |  |
| Basal ganglia infarction | 0.041 (0.001, 0.228) |  |
| Benign ear neoplasm | 0.041 (0.001, 0.228) |  |
| Benign lung neoplasm | 0.041 (0.001, 0.228) |  |
| Benign neoplasm of eyelid | 0.041 (0.001, 0.228) |  |
| Benign renal neoplasm | 0.041 (0.001, 0.228) |  |
| Biopsy breast normal | 0.041 (0.001, 0.228) |  |
| Biopsy kidney | 0.041 (0.001, 0.228) |  |
| Bladder cyst | 0.041 (0.001, 0.228) |  |
| Blindness transient | 0.041 (0.001, 0.228) |  |
| Blindness unilateral | 0.041 (0.001, 0.228) |  |
| Blood creatine increased | 0.041 (0.001, 0.228) |  |
| Blood homocysteine increased | 0.041 (0.001, 0.228) |  |
| Blood immunoglobulin A increased | 0.041 (0.001, 0.228) |  |
| Blood immunoglobulin M decreased | 0.041 (0.001, 0.228) |  |
| Blood loss anemia | 0.041 (0.001, 0.229) |  |
| Blood pressure fluctuation | 0.041 (0.001, 0.228) |  |
| Blood thyroid-stimulating hormone decreased | 0.041 (0.001, 0.228) |  |
| Blood triglycerides increased | 0.041 (0.001, 0.229) |  |
| Blood uric acid increased | 0.041 (0.001, 0.228) |  |
| Body tinea | 0.041 (0.001, 0.228) |  |
| Bone cyst | 0.041 (0.001, 0.229) |  |
| Bone loss | 0.041 (0.001, 0.229) |  |
| Breast calcifications | 0.041 (0.001, 0.228) |  |
| Breast cyst | 0.041 (0.001, 0.228) |  |
| Breast discharge | 0.041 (0.001, 0.228) |  |
| Breast discomfort | 0.041 (0.001, 0.228) |  |
| Breast inflammation | 0.041 (0.001, 0.228) |  |
| Breast necrosis | 0.041 (0.001, 0.228) |  |
| Breast pain | 0.041 (0.001, 0.228) |  |
| Bronchial irritation | 0.041 (0.001, 0.228) |  |
| Bronchiectasis | 0.041 (0.001, 0.228) |  |
| Broncholithiasis | 0.041 (0.001, 0.228) |  |
| Bruxism | 0.041 (0.001, 0.228) |  |
| C-reactive protein abnormal | 0.041 (0.001, 0.228) |  |
| *Campylobacter* gastroenteritis | 0.041 (0.001, 0.228) |  |
| Carbuncle | 0.041 (0.001, 0.228) |  |
| Cardiac valve disease | 0.041 (0.001, 0.228) |  |
| Central nervous system lesion | 0.041 (0.001, 0.228) |  |
| Cerebellar stroke | 0.041 (0.001, 0.229) |  |
| Cerebral artery embolism | 0.041 (0.001, 0.228) |  |
| Cerebral artery stenosis | 0.041 (0.001, 0.229) |  |
| Cerebrospinal fluid leakage | 0.041 (0.001, 0.228) |  |
| Chemical peel of skin | 0.041 (0.001, 0.228) |  |
| Chest injury | 0.041 (0.001, 0.228) |  |
| Chondromalacia | 0.041 (0.001, 0.229) |  |
| Chromaturia | 0.041 (0.001, 0.228) |  |
| Chronic gastritis | 0.041 (0.001, 0.228) |  |
| Chronic hepatitis | 0.041 (0.001, 0.228) |  |
| Claustrophobia | 0.041 (0.001, 0.228) |  |
| Closed fracture manipulation | 0.041 (0.001, 0.228) |  |
| *Clostridium difficile* colitis | 0.041 (0.001, 0.228) |  |
| Cogwheel rigidity | 0.041 (0.001, 0.228) |  |
| Compartment syndrome | 0.041 (0.001, 0.228) |  |
| Complication associated with device | 0.041 (0.001, 0.229) |  |
| Congenital cystic kidney disease | 0.041 (0.001, 0.228) |  |
| Congestive cardiomyopathy | 0.041 (0.001, 0.228) |  |
| Corneal degeneration | 0.041 (0.001, 0.228) |  |
| Coronavirus infection | 0.041 (0.001, 0.228) |  |
| Crepitations | 0.041 (0.001, 0.229) |  |
| Cyst removal | 0.041 (0.001, 0.228) |  |
| Dermatochalasis | 0.041 (0.001, 0.228) |  |
| Developmental hip dysplasia | 0.041 (0.001, 0.228) |  |
| Diabetes mellitus inadequate control | 0.041 (0.001, 0.228) |  |
| Diastolic dysfunction | 0.041 (0.001, 0.229) |  |
| Diverticular perforation | 0.041 (0.001, 0.229) |  |
| Drug intolerance | 0.041 (0.001, 0.228) |  |
| Duodenal ulcer hemorrhage | 0.041 (0.001, 0.228) |  |
| Dysentery | 0.041 (0.001, 0.228) |  |
| Dyskinesia | 0.041 (0.001, 0.229) |  |
| Dysphoria | 0.041 (0.001, 0.229) |  |
| ECG signs of myocardial ischemia | 0.041 (0.001, 0.229) |  |
| Ear canal stenosis | 0.041 (0.001, 0.228) |  |
| Ear congestion | 0.041 (0.001, 0.228) |  |
| Effusion | 0.041 (0.001, 0.228) |  |
| Electric shock | 0.041 (0.001, 0.229) |  |
| Electrocardiogram PR prolongation | 0.041 (0.001, 0.228) |  |
| Embolic stroke | 0.041 (0.001, 0.228) |  |
| Endocrine ophthalmopathy | 0.041 (0.001, 0.228) |  |
| Enlarged uvula | 0.041 (0.001, 0.228) |  |
| Enterocolitis viral | 0.041 (0.001, 0.228) |  |
| Eructation | 0.041 (0.001, 0.228) |  |
| Erythema multiforme | 0.041 (0.001, 0.228) |  |
| Eye infection bacterial | 0.041 (0.001, 0.228) |  |
| Eye movement disorder | 0.041 (0.001, 0.228) |  |
| Eyelid irritation | 0.041 (0.001, 0.228) |  |
| Eyelid edema | 0.041 (0.001, 0.228) |  |
| Feces hard | 0.041 (0.001, 0.228) |  |
| Femoroacetabular impingement | 0.041 (0.001, 0.228) |  |
| Fibrin D-dimer increased | 0.041 (0.001, 0.228) |  |
| Fibrosis | 0.041 (0.001, 0.228) |  |
| Fluid retention | 0.041 (0.001, 0.228) |  |
| Foreign body in eye | 0.041 (0.001, 0.229) |  |
| Foreign body in gastrointestinal tract | 0.041 (0.001, 0.229) |  |
| Fructose intolerance | 0.041 (0.001, 0.228) |  |
| Gangrene | 0.041 (0.001, 0.228) |  |
| Gastric mucosa erythema | 0.041 (0.001, 0.228) |  |
| Gastrointestinal sounds abnormal | 0.041 (0.001, 0.228) |  |
| Genital infection bacterial | 0.041 (0.001, 0.229) |  |
| Giant cell arteritis | 0.041 (0.001, 0.228) |  |
| Gingival erosion | 0.041 (0.001, 0.228) |  |
| Glioblastoma | 0.041 (0.001, 0.228) |  |
| Glossitis | 0.041 (0.001, 0.228) |  |
| Grief reaction | 0.041 (0.001, 0.228) |  |
| Grip strength decreased | 0.041 (0.001, 0.228) |  |
| Hemangioma of bone | 0.041 (0.001, 0.229) |  |
| Hemarthrosis | 0.041 (0.001, 0.228) |  |
| Hemoglobin increased | 0.041 (0.001, 0.228) |  |
| Hemorrhage intracranial | 0.041 (0.001, 0.228) |  |
| Hemorrhage subcutaneous | 0.041 (0.001, 0.228) |  |
| Hemorrhagic cyst | 0.041 (0.001, 0.228) |  |
| Hemorrhoids thrombosed | 0.041 (0.001, 0.228) |  |
| Hair color changes | 0.041 (0.001, 0.229) |  |
| Hallucination, visual | 0.041 (0.001, 0.228) |  |
| Hamartoma | 0.041 (0.001, 0.228) |  |
| Hearing aid user | 0.041 (0.001, 0.228) |  |
| *Helicobacter* test positive | 0.041 (0.001, 0.228) |  |
| Hepatic cyst ruptured | 0.041 (0.001, 0.228) |  |
| Hepatitis A | 0.041 (0.001, 0.229) |  |
| Hypercoagulation | 0.041 (0.001, 0.228) |  |
| Hyperthyroidism | 0.041 (0.001, 0.228) |  |
| Hyperaldosteronism | 0.041 (0.001, 0.228) |  |
| Hypothermia | 0.041 (0.001, 0.228) |  |
| Ileus | 0.041 (0.001, 0.228) |  |
| Implant site infection | 0.041 (0.001, 0.228) |  |
| Incarcerated inguinal hernia | 0.041 (0.001, 0.228) |  |
| Infected bite | 0.041 (0.001, 0.228) |  |
| Infected skin ulcer | 0.041 (0.001, 0.229) |  |
| Influenza A virus test positive | 0.041 (0.001, 0.228) |  |
| Infusion site induration | 0.041 (0.001, 0.228) |  |
| Inguinal hernia strangulated | 0.041 (0.001, 0.228) |  |
| Injection site pain | 0.041 (0.001, 0.228) |  |
| Intervertebral disc operation | 0.041 (0.001, 0.228) |  |
| Intestinal ischaemia | 0.041 (0.001, 0.228) |  |
| Intra-abdominal haematoma | 0.041 (0.001, 0.228) |  |
| Invasive breast carcinoma | 0.041 (0.001, 0.228) |  |
| Joint hyperextension | 0.041 (0.001, 0.229) |  |
| Joint noise | 0.041 (0.001, 0.228) |  |
| Keloid scar | 0.041 (0.001, 0.228) |  |
| Labile hypertension | 0.041 (0.001, 0.228) |  |
| Lacrimation disorder | 0.041 (0.001, 0.229) |  |
| Lacunar stroke | 0.041 (0.001, 0.229) |  |
| Language disorder | 0.041 (0.001, 0.229) |  |
| Left atrial dilatation | 0.041 (0.001, 0.228) |  |
| Lens capsulotomy | 0.041 (0.001, 0.228) |  |
| Lentigo | 0.041 (0.001, 0.228) |  |
| Lentigo maligna | 0.041 (0.001, 0.228) |  |
| Limb discomfort | 0.041 (0.001, 0.228) |  |
| Lip blister | 0.041 (0.001, 0.228) |  |
| Lip dry | 0.041 (0.001, 0.229) |  |
| Lip infection | 0.041 (0.001, 0.229) |  |
| Liposuction | 0.041 (0.001, 0.228) |  |
| Livedo reticularis | 0.041 (0.001, 0.229) |  |
| Lower respiratory tract infection bacterial | 0.041 (0.001, 0.228) |  |
| Lumbar vertebral fracture | 0.041 (0.001, 0.228) |  |
| Lung carcinoma cell type unspecified stage IV | 0.041 (0.001, 0.228) |  |
| Lung neoplasm malignant | 0.041 (0.001, 0.228) |  |
| Lymphocyte count decreased | 0.041 (0.001, 0.229) |  |
| Lymphoma | 0.041 (0.001, 0.228) |  |
| Lymphoplasmacytoid lymphoma/immunocytoma | 0.041 (0.001, 0.228) |  |
| Macular detachment | 0.041 (0.001, 0.228) |  |
| Macular hole | 0.041 (0.001, 0.228) |  |
| Malignant melanoma stage I | 0.041 (0.001, 0.228) |  |
| Malpositioned teeth | 0.041 (0.001, 0.228) |  |
| Medical device site photosensitivity reaction | 0.041 (0.001, 0.228) |  |
| Medical device site reaction | 0.041 (0.001, 0.228) |  |
| Mental impairment | 0.041 (0.001, 0.229) |  |
| Metabolic encephalopathy | 0.041 (0.001, 0.228) |  |
| Metamorphopsia | 0.041 (0.001, 0.228) |  |
| Metastases to bone | 0.041 (0.001, 0.228) |  |
| Migraine with aura | 0.041 (0.001, 0.228) |  |
| Milia | 0.041 (0.001, 0.229) |  |
| Mixed deafness | 0.041 (0.001, 0.228) |  |
| Monoclonal B-cell lymphocytosis | 0.041 (0.001, 0.228) |  |
| Motion sickness | 0.041 (0.001, 0.229) |  |
| Mouth injury | 0.041 (0.001, 0.228) |  |
| Muscle atrophy | 0.041 (0.001, 0.228) |  |
| Muscle twitching | 0.041 (0.001, 0.228) |  |
| Myelomalacia | 0.041 (0.001, 0.228) |  |
| Myelopathy | 0.041 (0.001, 0.228) |  |
| Myoclonus | 0.041 (0.001, 0.228) |  |
| Nail discoloration | 0.041 (0.001, 0.229) |  |
| Nail infection | 0.041 (0.001, 0.229) |  |
| Nail injury | 0.041 (0.001, 0.229) |  |
| Nail operation | 0.041 (0.001, 0.228) |  |
| Nasal discomfort | 0.041 (0.001, 0.228) |  |
| Nasal herpes | 0.041 (0.001, 0.228) |  |
| Nasal inflammation | 0.041 (0.001, 0.229) |  |
| Nasal polyps | 0.041 (0.001, 0.228) |  |
| Nerve injury | 0.041 (0.001, 0.228) |  |
| Nervous system disorder | 0.041 (0.001, 0.228) |  |
| Neutropenia | 0.041 (0.001, 0.228) |  |
| Non-small cell lung cancer | 0.041 (0.001, 0.228) |  |
| Normal pressure hydrocephalus | 0.041 (0.001, 0.228) |  |
| Normocytic anemia | 0.041 (0.001, 0.228) |  |
| Nuchal rigidity | 0.041 (0.001, 0.228) |  |
| Occult blood | 0.041 (0.001, 0.228) |  |
| Esophageal compression | 0.041 (0.001, 0.228) |  |
| Esophageal irritation | 0.041 (0.001, 0.228) |  |
| Esophageal ulcer | 0.041 (0.001, 0.228) |  |
| Esophageal ulcerative | 0.041 (0.001, 0.228) |  |
| Optic disc drusen | 0.041 (0.001, 0.228) |  |
| Oral surgery | 0.041 (0.001, 0.228) |  |
| Osteosarcoma | 0.041 (0.001, 0.229) |  |
| Pancreatic carcinoma metastatic | 0.041 (0.001, 0.228) |  |
| Pancreatic carcinoma stage III | 0.041 (0.001, 0.228) |  |
| Pancreatic mass | 0.041 (0.001, 0.228) |  |
| Papillary thyroid cancer | 0.041 (0.001, 0.228) |  |
| Papilloma | 0.041 (0.001, 0.228) |  |
| Parainfluenza virus infection | 0.041 (0.001, 0.228) |  |
| Parathyroidectomy | 0.041 (0.001, 0.228) |  |
| Parkinsonian rest tremor | 0.041 (0.001, 0.228) |  |
| Pelvic discomfort | 0.041 (0.001, 0.228) |  |
| Pelvic pain | 0.041 (0.001, 0.228) |  |
| Peripheral nerve neurostimulation | 0.041 (0.001, 0.229) |  |
| Peritonsillar abscess | 0.041 (0.001, 0.228) |  |
| Persistent depressive disorder | 0.041 (0.001, 0.228) |  |
| Persistent postural-perceptual dizziness | 0.041 (0.001, 0.228) |  |
| Petechiae | 0.041 (0.001, 0.229) |  |
| Petrositis | 0.041 (0.001, 0.228) |  |
| Phlebitis superficial | 0.041 (0.001, 0.228) |  |
| Pleurisy | 0.041 (0.001, 0.228) |  |
| Pneumonia aspiration | 0.041 (0.001, 0.228) |  |
| Pneumonitis | 0.041 (0.001, 0.228) |  |
| Polycythemia | 0.041 (0.001, 0.228) |  |
| Polydipsia | 0.041 (0.001, 0.229) |  |
| Postoperative thrombosis | 0.041 (0.001, 0.229) |  |
| Precancerous condition | 0.041 (0.001, 0.228) |  |
| Procedural complication | 0.041 (0.001, 0.228) |  |
| Procedural hypertension | 0.041 (0.001, 0.228) |  |
| Protein C deficiency | 0.041 (0.001, 0.228) |  |
| Protein S decreased | 0.041 (0.001, 0.228) |  |
| Protein urine present | 0.041 (0.001, 0.229) |  |
| Proteinuria | 0.041 (0.001, 0.228) |  |
| Pruritus genital | 0.041 (0.001, 0.228) |  |
| Pulmonary calcification | 0.041 (0.001, 0.228) |  |
| Pulmonary granuloma | 0.041 (0.001, 0.228) |  |
| Pulmonary edema | 0.041 (0.001, 0.228) |  |
| Pulmonary valve incompetence | 0.041 (0.001, 0.229) |  |
| Radiation proctitis | 0.041 (0.001, 0.228) |  |
| Red blood cell count decreased | 0.041 (0.001, 0.229) |  |
| Renal hamartoma | 0.041 (0.001, 0.228) |  |
| Renal injury | 0.041 (0.001, 0.228) |  |
| Respiratory fume inhalation disorder | 0.041 (0.001, 0.229) |  |
| Respiratory tract congestion | 0.041 (0.001, 0.228) |  |
| Resting tremor | 0.041 (0.001, 0.228) |  |
| Retinal artery occlusion | 0.041 (0.001, 0.229) |  |
| Retroperitoneal hemorrhage | 0.041 (0.001, 0.228) |  |
| Right ventricular failure | 0.041 (0.001, 0.228) |  |
| Rocky mountain spotted fever | 0.041 (0.001, 0.228) |  |
| Root canal infection | 0.041 (0.001, 0.228) |  |
| Sacral pain | 0.041 (0.001, 0.228) |  |
| Scapholunate dissociation | 0.041 (0.001, 0.228) |  |
| Scapula fracture | 0.041 (0.001, 0.228) |  |
| Scleral hemorrhage | 0.041 (0.001, 0.229) |  |
| Scrotal hemorrhage | 0.041 (0.001, 0.228) |  |
| Seborrhea | 0.041 (0.001, 0.228) |  |
| Sinonasal obstruction | 0.041 (0.001, 0.228) |  |
| Sinus polyp | 0.041 (0.001, 0.229) |  |
| Sjogren's syndrome | 0.041 (0.001, 0.228) |  |
| Skin hyperpigmentation | 0.041 (0.001, 0.228) |  |
| Skin neoplasm excision | 0.041 (0.001, 0.228) |  |
| Sleep deficit | 0.041 (0.001, 0.228) |  |
| Sneezing | 0.041 (0.001, 0.228) |  |
| Soft tissue swelling | 0.041 (0.001, 0.228) |  |
| Spinal synovial cyst | 0.041 (0.001, 0.228) |  |
| Staphylococcal skin infection | 0.041 (0.001, 0.228) |  |
| Status epilepticus | 0.041 (0.001, 0.228) |  |
| Steatohepatitis | 0.041 (0.001, 0.228) |  |
| Stenosis | 0.041 (0.001, 0.229) |  |
| Strabismus correction | 0.041 (0.001, 0.228) |  |
| Streptococcal sepsis | 0.041 (0.001, 0.228) |  |
| Subdural hemorrhage | 0.041 (0.001, 0.228) |  |
| Subdural hygroma | 0.041 (0.001, 0.229) |  |
| Swelling of eyelid | 0.041 (0.001, 0.228) |  |
| Synovitis | 0.041 (0.001, 0.229) |  |
| Systemic mycosis | 0.041 (0.001, 0.229) |  |
| Tachyarrhythmia | 0.041 (0.001, 0.228) |  |
| Tendon calcification | 0.041 (0.001, 0.228) |  |
| Terminal insomnia | 0.041 (0.001, 0.228) |  |
| Therapeutic procedure | 0.041 (0.001, 0.228) |  |
| Thoracic radiculopathy | 0.041 (0.001, 0.228) |  |
| Thrombophlebitis | 0.041 (0.001, 0.229) |  |
| Thyroid cancer | 0.041 (0.001, 0.228) |  |
| Tibia fracture | 0.041 (0.001, 0.228) |  |
| Tricuspid valve prolapse | 0.041 (0.001, 0.228) |  |
| Trunk injury | 0.041 (0.001, 0.228) |  |
| Tunnel vision | 0.041 (0.001, 0.229) |  |
| Upper respiratory tract infection bacterial | 0.041 (0.001, 0.228) |  |
| Upper respiratory tract irritation | 0.041 (0.001, 0.228) |  |
| Ureterolithiasis | 0.041 (0.001, 0.229) |  |
| Urethral disorder | 0.041 (0.001, 0.228) |  |
| Uveitis | 0.041 (0.001, 0.229) |  |
| VIth nerve paralysis | 0.041 (0.001, 0.228) |  |
| Vaccination site joint pain | 0.041 (0.001, 0.228) |  |
| Vaccination site pruritus | 0.041 (0.001, 0.228) |  |
| Vascular parkinsonism | 0.041 (0.001, 0.228) |  |
| Venomous sting | 0.041 (0.001, 0.228) |  |
| Venous thrombosis limb | 0.041 (0.001, 0.229) |  |
| Vestibular migraine | 0.041 (0.001, 0.228) |  |
| Viral labyrinthitis | 0.041 (0.001, 0.229) |  |
| Viral tonsillitis | 0.041 (0.001, 0.228) |  |
| Vitreous hemorrhage | 0.041 (0.001, 0.229) |  |

Data are presented as incidence rate per 100 PY (95% CI).

*Male-specific event (N=234).

^†^Female-specific event (N=357).

CI = confidence interval; COVID-19 = coronavirus disease 2019; ECG = electrocardiogram; IR = incidence rate; N = number of patients at risk for each; PY = person-years; SARS-CoV = severe acute respiratory syndrome coronavirus 2; TEAE = treatment-emergent adverse event.

**Supplementary Table 3. Participants in the placebo arm during the double-blind period of the A4 study with suicidal ideation, suicidal behavior, and self-injurious behavior without suicidal intent, based on the C-SSRS during treatment (safety population)**

| **Events during treatment, n (%)** | **Placebo**  **(N=583)** | **Incidence Rate  per 100 PY (95% CI)** |
| --- | --- | --- |
| Suicidal Ideation* | 44 (7.5) | 1.879 (1.366, 2.523) |
| Wish to be dead | 28 (4.8) | 1.171 (0.778, 1.693) |
| Non-specific active suicidal thoughts | 21 (3.6) | 0.881 (0.545, 1.347) |
| Active suicidal ideation with any methods (not plan) without intent to act | 8 (1.4) | 0.330 (0.143, 0.651) |
| Active suicidal ideation with some intent to act, without specific plan | 2 (0.3) | 0.082 (0.010, 0.296) |
| Active suicidal ideation with specific plan and intent | 2 (0.3) | 0.082 (0.010, 0.297) |
| Suicidal behavior^†^ | 6 (1.0) | 0.247 (0.091, 0.538) |
| Preparatory acts or behavior | 5 (0.9) | 0.206 (0.067, 0.480) |
| Aborted attempt | 1 (0.2) | 0.041 (0.001, 0.228) |
| Interrupted attempt | 0 |  |
| Non-fatal suicide attempt | 0 |  |
| Completed suicide | 0 |  |
| Suicidal ideation or behavior^‡^ | 47 (8.1) | 2.017 (1.482, 2.682) |
| Self-injurious behavior without suicidal intent | 0 |  |

Data are presented as n (%) unless otherwise indicated.

C-SSRS = Columbia Suicide Severity Rating Scale; CI = confidence interval; N = number of patients in the analysis population with at least one post-baseline C-SSRS assessment; n = the number of patients who experience the event at least once during treatment; PY = person-years.

* n and (%) refer to the number and percent of patients, respectively, who experience any one of the five listed suicidal ideation events at least once during treatment.

^†^ n and (%) refer to the number and percent of patients, respectively, who experience any one of the five listed suicidal behavior events at least once during treatment.

^‡^ n and (%) refer to the number and percent of patients, respectively, who experience any one of the ten suicidal ideation or behavior events at least once during treatment.

**Supplementary Table 4. Treatment-emergent ECG abnormalities among participants in the placebo arm during the double-blind period of the A4 study (safety population)**

| **ECG Parameters** | **Placebo** | | |
| --- | --- | --- | --- |
|  | N | n (%) | Incidence Rate  per 100 PY (95% CI) |
| PR Interval |  |  |  |
| Low (≤120 msec) | 573 | 8 (1.4) | 0.331 (0.143, 0.652) |
| High (≥220 msec) | 542 | 34 (6.3) | 1.439 (0.997, 2.012) |
| QRS Complex |  |  |  |
| Low (≤50 msec) | 581 | 0 (0.0) |  |
| High (≥120 msec) | 540 | 18 (3.3) | 0.747 (0.443, 1.181) |
| QT Interval |  |  |  |
| Low (no lower limit) | 0 | 0 |  |
| High (≥450 msec) | 549 | 64 (11.7) | 2.818 (2.170, 3.598) |
| Mean heart rate |  |  |  |
| Low (≤40 bpm) | 550 | 71 (12.9) | 3.167 (2.473, 3.995) |
| High (≥120 bpm) | 581 | 4 (0.7) | 0.164 (0.045, 0.421) |

Percentages were calculated using N as the denominator for each ECG parameter.

A treatment-emergent abnormal measurement is defined as a change from normal during the baseline period to abnormal at a post-baseline visit. All scheduled and unscheduled results are included.

Safety analysis set includes participants having taken the study drug.

bpm = beats per minute; CI = confidence interval; ECG = electrocardiogram; msec = milliseconds; n = number of participants with an abnormal post-baseline result in the specified category; N = number of participants with a normal (ie, not low if calculating “Treatment-Emergent low” and not high if calculating “Treatment-Emergent high”) baseline and at least one non-missing post-baseline value; PY = person-years.

**Supplementary Table 5. Most significant baseline factors associated with ARIA-H among participants in the placebo arm during the double-blind period of the A4 study (safety population)**

| **Baseline factors associated with ARIA-H** | **OR (95% CI)** | **P-value** | **Adjusted P-value** |
| --- | --- | --- | --- |
| Microhemorrhages, 0 vs. ≥1 | 349.9 (247.6–494.4) | <0.001 | <0.001 |
| APOE ɛ4, non-carrier vs homozygote | 3.4 (2.4–4.7) | <0.001 | <0.001 |
| Platelets* | 0.5 (0.4–0.8) | 0.005 | 0.011 |
| Sex, female vs male | 1.5 (1.1–2.1) | 0.018 | 0.019 |
| Plasma P-tau217* | 1.7 (1.2–2.4) | 0.019 | 0.038 |
| Amyloid PET centiloid* | 1.6 (1.1–2.6) | 0.030 | 0.061 |
| Age* | 1.6 (1.1–2.3) | 0.032 | 0.063 |
| Mean arterial pressure* | 1.6 (1.1–2.2) | 0.034 | 0.067 |
| Lateral ventricular volume* | 1.6 (1.1–2.2) | 0.042 | 0.084 |

APOE = apolipoprotein E; ARIA-H = amyloid-related imaging abnormalities–hemosiderin deposition; CI = confidence interval; OR = odds ratio; PET = positron emission tomography.

* Continuous values for platelets, plasma P-tau217, amyloid PET, age, mean arterial pressure, and lateral ventricular volume were categorized into tertiles; the ORs represent a comparison of the highest tertile to the lowest tertile for all continuous baseline factors except amyloid PET, where the OR represents a comparison of the middle tertile to the lowest tertile.
